# Two routes for tyrosol production by metabolic engineering of *Corynebacterium glutamicum*

**DOI:** 10.1186/s13068-025-02641-6

**Published:** 2025-04-05

**Authors:** Nora Junker, Sara-Sophie Poethe, Volker F. Wendisch

**Affiliations:** Genetics of Prokaryotes, Faculty of Biology and Center for Biotechnology (CeBiTec), Bielefeld University, Bielefeld, Germany

**Keywords:** *Corynebacterium glutamicum*, Tyrosol overproduction, Pathway comparison, Metabolic engineering

## Abstract

**Background:**

The phenolic compound tyrosol is widely used in the pharmaceutical industry, owing to its beneficial effects on human health and its use as a precursor for key pharmaceuticals, including β_1_-receptor blockers. Tyrosol can be found in olive oil, but despite its natural biosynthesis in plants, low extraction efficiencies render microbial production a more viable alternative.

**Results:**

Here, we engineered the l-tyrosine overproducing *Corynebacterium glutamicum* strain AROM3 for the de novo production of tyrosol. Two routes were established and compared: one via 4-OH-phenylpyruvate as intermediate and the other via tyramine. We initially expected the first route to require heterologous expression of a prephenate dehydrogenase gene, given that *C. glutamicum* lacks this enzymatic function. However, heterologous expression of *ARO10* from *Saccharomyces cerevisiae* (*ARO10*_*Sc*_), which encodes a phenylpyruvate decarboxylase, was sufficient to establish tyrosol production in strain AROM3. We identified that 4-OH-phenylpyruvate is synthesized from l-tyrosine by native aminotransferases, which is subsequently decarboxylated by Aro10_*Sc*_*,* and reduced to tyrosol by native alcohol dehydrogenases, leading to a titer of 9.4 ± 1.1 mM (1.30 ± 0.15 g/L). We identified the furfural dehydrogenase FudC as major enzyme involved in this pathway, as its gene deletion reduced tyrosol production by 75%. Given the instability of 4-OH-phenylpyruvate, the synthesis of tyrosol via the stable intermediate tyramine was pursued via the second route. Decarboxylation of l-tyrosine followed by oxidative deamination was accomplished by overexpression of the l-tyrosine decarboxylase gene *tdc* from *Levilactobacillus brevis* (*tdc*_*Lb*_) and the tyramine oxidase gene *tyo* from *Kocuria rhizophila* (*tyo*_*Kr*_). Using this route, tyrosol production was increased by 44% compared to the route via 4-OH-phenylpyruvate. With a division of labor approach by co-cultivating l-tyrosine producing strains that either express *tdc*_*Lb*_ or *tyo*_*Kr*_, the highest titer of 14.1 ± 0.3 mM (1.95 ± 0.04 g/L) was achieved.

**Conclusions:**

This study demonstrates the potential of endotoxin-free *C. glutamicum* as production host for the l-tyrosine-derived product tyrosol. Due to its l-arogenate pathway for l-tyrosine synthesis, the unstable 4-OH-phenylpyruvate could be excluded as intermediate in the Tdc–Tyo pathway, outcompeting the most often utilized production route via phenylpyruvate decarboxylases.

**Supplementary Information:**

The online version contains supplementary material available at 10.1186/s13068-025-02641-6.

## Introduction

Oxidative damage plays a significant role in the development of various diseases, including Alzheimer's, Parkinson's, cardiovascular diseases, and cancer [1]. The phenolic compound tyrosol (2-[4-hydroxyphenyl]-ethanol) can protect tissues and cells from oxidative stress due to regulatory effects [2, 3]. Several studies demonstrated the beneficial impact of the bioactive compound tyrosol on human health, including reducing the risk of cardiovascular or Alzheimer's diseases [4–6]. In addition, tyrosol serves as a precursor for commercially available pharmaceutical agents and, thus, finds extensive use in the pharmaceutical industry. Tyrosol is, for example, employed in the synthesis of β_1_-receptor blockers [7, 8] and salidroside [9], both used to treat cardiovascular diseases [10, 11].

While different chemical synthesis methods are available for the production of tyrosol, e.g., from 4-bromophenol or 2-phenylethanol [12], their main drawbacks are expensive precursors and harsh process conditions. Natural sources of tyrosol such as olive oil offer an alternative supply. Extraction from olives or from olive oil mill wastewater, a byproduct of olive oil production [13], shows low profitability due to low concentrations of tyrosol in plant sources (e.g., 43–68 mg/kg in olive oil [14]), that require complex extraction procedures. Due to the drawbacks of existing processes, there is a growing focus on low-cost and eco-friendly biosynthesis using microorganisms.

Tyrosol can be produced via different routes, which are related to the l-tyrosine synthesis pathway. For one, l-tyrosine itself can be used in biotransformation as a precursor for tyrosol synthesis, which can be decarboxylated to tyramine by an l-tyrosine decarboxylase (Tdc) and subsequently deaminated by a tyramine oxidase (Tyo). The resulting 4-OH-phenylacetaldyhyde can finally be reduced to tyrosol by an alcohol dehydrogenase (Adh) [15]. The immediate precursor 4-OH-phenylacetaldyhyde can, however, also be synthesized by decarboxylation of 4-OH-phenylpyruvate, which is a precursor of l-tyrosine in many microorganisms or can be derived from l-tyrosine as degradation product [16].

Yeasts, such as *Saccharomyces cerevisiae* and *Candida albicans,* naturally produce tyrosol as a quorum sensing molecule via the latter pathway [17, 18]. Consequently, they are already equipped with the required enzymatic pathway and have been further engineered to improve the production of tyrosol, resulting in titers up to 1 g/L (7 mM) [19–21]. As bacteria, such as *Escherichia coli,* generally have a higher specific productivity, resulting in lower process costs [22], their genetic engineering for tyrosol production has received considerable attention. *E. coli* Δ*feaB* strains with plasmid-based expression of the phenylpyruvate decarboxylase gene *ARO10*_*Sc*_ or expression of *tdc* and *tyo*, from *Papaver somniferum* and *Micrococcus luteus*, produced 0.41 mM and 0.5 mM tyrosol, respectively [15, 23]. With further metabolic engineering, titers were increased to 4–9 mM tyrosol in shake flask cultivations [23–25]. Besides the pathway using Aro10_*Sc*_ or Tdc and Tyo, other more complex routes have been tried including 5 enzymatically catalyzed steps from l-tyrosine to tyrosol, albeit titers were comparably low [26]. A major challenge in large-scale production, however, is the removal of *E. coli* lipopolysaccharides, also referred to as endotoxin, which are part of the outer membrane of *E. coli*. This potent immunostimulant induces a pyrogenic response in humans, leading to symptoms of inflammation, ranging from fever up to septic shock [27].

Endotoxin-free *Corynebacterium glutamicum* offers several advantages, most notably its established use for industrial production of generally recognized as safe (GRAS) food ingredients, including amino acid production at the million-ton scale [28]. Its previous engineering for l-tyrosine overproduction makes *C.* *glutamicum* a superior candidate for producing the l-tyrosine derivative tyrosol. Overproduction of l-tyrosine was achieved by metabolic engineering, which included the genomic integration of the gene encoding a feedback-inhibition-resistant mutant of the 3-deoxy-d-arabinoheptulosonate-7-phosphate (DAHP) synthase from *E. coli* (*aroG*_*Ec*_^fbr^). In addition, start codon exchanges from ATG to the less frequently used TTG in the genes *pheA* and *trpE* were introduced to reduce the competing biosynthesis of the amino acids l-phenylalanine and l-tryptophan. The derived strain *C.* *glutamicum* AROM3 produced 17 mM (3.1 g/L) l-tyrosine [29]. To broaden feedstock flexibility [30], l-tyrosine and tyramine were also produced from xylose and orange peel hydrolysate [29, 31, 32].

Here, we compared for the first time both routes for tyrosol production in the same background strain. In this study, we constructed tyrosol producing *C.* *glutamicum* strains using either 4-OH-phenylpyruvate or tyramine as precursor. With both pathways, we could successfully demonstrate the de novo production of tyrosol in recombinant *C.* *glutamicum* at g/L scale.

## Results

### Physiological response of *C. glutamicum* to tyrosol

Tyrosol can be synthesized from the amino acid l-tyrosine or from its precursor prephenate. Therefore, *C.* *glutamicum* AROM3, a genome-reduced strain, which we had previously engineered for the overproduction of l-tyrosine [29], was chosen as basis.

**Fig. 1:**
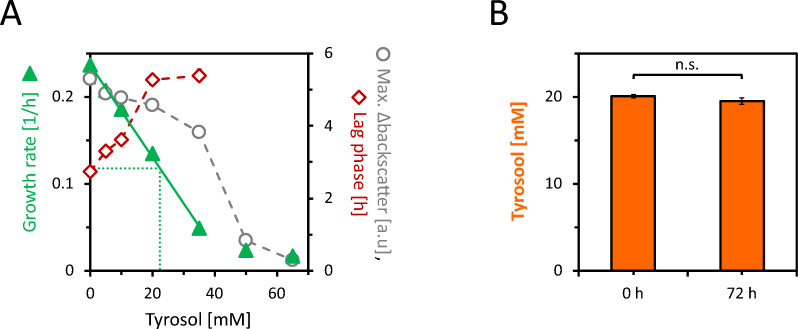
Growth impairment of *C.* *glutamicum* AROM3 due to tyrosol supplementation (**A**) and tyrosol degradation test (**B**). AROM3 was cultivated in 1 mL CGXII minimal medium containing 40 g/L glucose, and varying tyrosol concentrations ranging from 0 to 65 mM. The backscatter was measured in a BioLector cultivation system and used to calculate the specific growth rate (green triangles), maximum Δbackscatter (grey circles), and lag phase (red diamonds) for the AROM3 cultures exposed to different tyrosol concentrations (**A**). The tyrosol concentration leading to a half-maximum growth rate could be estimated from the regression line to be 22 mM, as indicated by the green dotted line. Tyrosol concentrations determined at the beginning and the end of cultivation in the cultures containing 20 mM tyrosol were not significantly different (n.s.: *p* > 0.05) according to calculation with a two-sided Student’s *t* test (**B**). Values and error bars represent means and standard deviations of triplicate cultivations

To evaluate the suitability of *C.* *glutamicum* AROM3 for tyrosol production, possible toxic effects of tyrosol and its potential degradation were tested. AROM3 was cultivated in the BioLector microcultivation system in CGXII minimal medium with 40 g/L glucose in the presence of 0–65 mM tyrosol for 72 h. Supplementation of tyrosol decreased the specific growth rate and biomass formation for all tested concentrations (Fig. 1A). With supplementation of 20 mM tyrosol (2.8 g/L), the growth rate of AROM3 was about half-maximal, and the lag phase almost doubled to 5.3 h. At 65 mM tyrosol, AROM3 was unable to grow. A K_i_ value of 22 mM was determined when growth rates were plotted against tyrosol concentrations (Fig. 1A). The observed toxic effect of tyrosol might be attributed to its inhibitory effect on bacterial ATP synthases [33]. It is noteworthy that AROM3 showed a considerably greater robustness to tyrosol than *E. coli*, which exhibited complete inhibition of growth at a tyrosol concentration of 25 mM [33].

In addition to the assessment of its toxic effects, tyrosol was quantified at the beginning and after 72 h of cultivation to test for its potential degradation by AROM3. The observation that the tyrosol concentrations did not decrease significantly during cultivation (Fig. 1B) indicated that tyrosol is not degraded by AROM3. Taken together, *C.* *glutamicum* AROM3 is suited for production of tyrosol to concentrations in the g/L scale.

### Production of tyrosol from 4-OH-phenylpyruvate

**Fig. 2:**
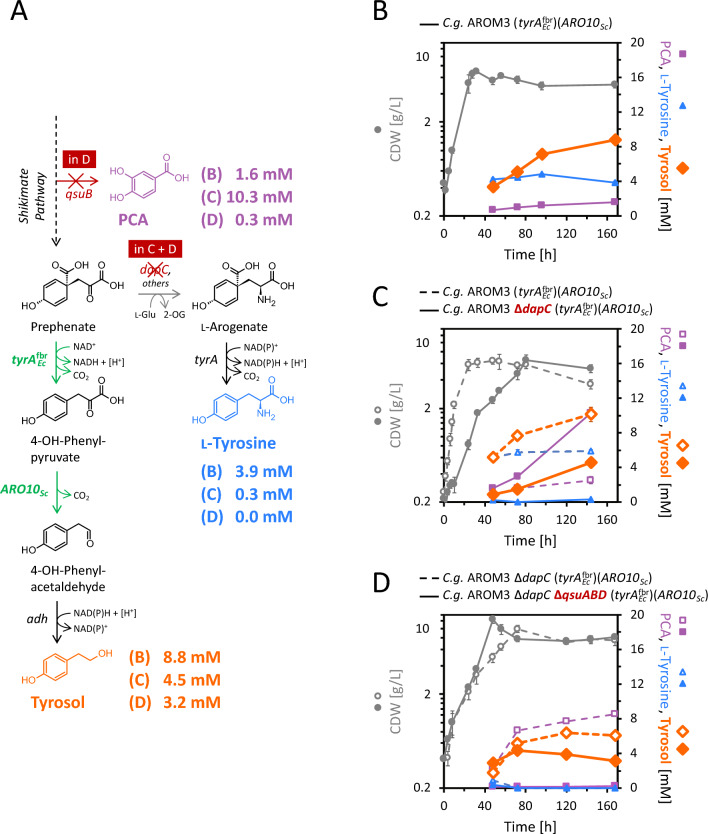
Tyrosol production with plasmid-based expression of *tyrA*_*Ec*_^fbr^ and *ARO10*_*Sc*_ in strain *C.* *glutamicum* AROM3 and derived deletion strains. **A** Tyrosol synthesis pathway with 4-OH-phenylpyruvate as intermediate via the overexpression of the heterologous genes *tyrA*_*Ec*_^fbr^ and *ARO10*_*Sc*_ (green), encoding a feedback resistant mutant of the bifunctional chorismate mutase/prephenate dehydrogenase from *E. coli* and phenylpyruvate decarboxylase from *S. cerevisiae*, respectively. Gene deletions of *qsuB* and *dapC,* encoding 3-dehydroshikimate dehydratase and *N*-succinyl-aminooxopimelate aminotransferase, respectively, are indicated by red crosses. Final production titers of PCA, l-tyrosine, and tyrosol are shown for the tested strains (indicated by B–D). Native enzymes (black) are encoded by *tyrA*: l-arogenate decarboxylase, *adh*: alcohol dehydrogenase. PCA: protocatechuate; l-Glu: l-glutamate; 2-OG: 2-oxoglutarate; 4-OH: 4-hydroxy. NAD(P): nicotinamide adenine dinucleotide (phosphate). **B–D** Growth (CDW) and production of PCA, l-tyrosine, and tyrosol for *C.* *glutamicum* AROM3 (*tyrA*_*Ec*_^fbr^)(*ARO10*_*Sc*_) (after 96 h, glucose was exhausted) (**B**), and its comparison to the respective *dapC* deletion strain (for which the glucose was exhausted after 144 h) (**C**), and further comparison to strain *C.* *glutamicum* AROM3 Δ*dapC* Δ*qsuABD* (*tyrA*_*Ec*_^fbr^)(*ARO10*_*Sc*_) (which had depleted glucose after 72 h) (**D**). Values and error bars represent means and standard deviations of triplicate shake flask cultivations

One option to synthesize tyrosol is via 4-OH-phenylpyruvate as a precursor. This metabolite is an intermediate in l-tyrosine synthesis in many microorganisms, including *E. coli* [34]*,* but not in *C.* *glutamicum*. *C.* *glutamicum* lacks a prephenate dehydrogenase (PDH, EC 1.3.1.12) for the synthesis of 4-OH-phenylpyruvate from prephenate and produces l-tyrosine via l-arogenate (pretyrosine) as an intermediate instead [35] (Fig. 2A). Therefore, we selected the gene encoding a feedback-resistant mutant of the bifunctional chorismate mutase/prephenate dehydrogenase from *E. coli* (TyrA_*Ec*_^fbr^) [36, 37] for expression in *C.* *glutamicum* AROM3. As TyrA_*Ec*_^fbr^ converts chorismate via prephenate to 4-OH-phenylpyruvate, functional expression in *C.* *glutamicum* AROM3 was tested by deletion of the native chorismate mutase gene *csm.* l-Tyrosine auxotrophy of this deletion strain AROM3 Δ*csm*, was successfully overcome by expression of *tyrA*_*Ec*_^fbr^ (Figure S1).

The synthesis of tyrosol from 4-OH-phenylpyruvate has for instance been described in the yeast *Saccharomyces cerevisiae* [16]. In the Ehrlich pathway, Aro10_*Sc*_ decarboxylates 4-OH-phenylpyruvate to 4-OH-phenylacetaldehyde [38]. The 2-oxoisovalerate decarboxylase KivD from *Lactococcus lactis* also showed activity for 4-OH-phenylpyruvate decarboxylation, however, in tyrosol production experiments with *E. coli*, Aro10 from *S. cerevisiae* was shown to be the better candidate [39]. Consequently, plasmid-based expression of the gene *ARO10*_*Sc*_, which was codon harmonized for *C.* *glutamicum*, was tested together with the expression of *tyrA*_*Ec*_^fbr^. It was anticipated that 4-OH-phenylacetaldehyde, which is formed by the combined action of TyrA_*Ec*_^fbr^ and Aro10_*Sc*_, is reduced to tyrosol by (a) native alcohol dehydrogenase(s) of *C.* *glutamicum*.

Upon shake flask cultivation in CGXII medium with 40 g/L glucose, AROM3 (*tyrA*_*Ec*_^fbr^) (*ARO10*_*Sc*_) produced 8.8 ± 0.3 mM tyrosol (equivalent to 1.21 ± 0.04 g/L) within 168 h (Fig. 2B), which was quantified by HPLC measurement and additionally verified by GC–MS analysis (Figure S2). Tyrosol was not detected in the supernatants of the parental strain AROM3 nor in the empty vector harboring strain AROM3 (*tyrA*_*Ec*_^fbr^) (EV2), which both accumulated l-tyrosine (Figure S3).

The competing product l-tyrosine was also accumulated by AROM3 (*tyrA*_*Ec*_^fbr^) (*ARO10*_*Sc*_). We previously demonstrated that deletion of the aminotransferase gene *dapC* in *C. glutamicum* wild type causes l-tyrosine bradytrophy and, thus, its involvement in the transamination of prephenate to l-arogenate in l-tyrosine biosynthesis [29]. Consequently, to lower l-tyrosine synthesis and reduce competition for the precursor prephenate, we deleted *dapC* in AROM3. Indeed, the l-tyrosine titer was decreased from 5.9 ± 0.2 mM for the parental strain to less than 0.3 mM (144 h) for strain AROM3 Δ*dapC* (*tyrA*_*Ec*_^fbr^) (*ARO10*_*Sc*_). However, tyrosol synthesis was not increased (Fig. 2C). Even with supplementation of l-tyrosine and l-lysine to boost growth of the bradytrophic *dapC* deletion strain, tyrosol production remained inferior to the parental strain (Figure S4B, C). Instead of increased tyrosol titers, the strain showed an accelerated production of a different metabolite derived from the shikimate pathway, which was identified to be protocatechuate (PCA) (3,4‐dihydroxybenzoate). Due to the deletion of *pcaHG*, encoding the protocatechuate 3,4-dioxygenase, PCA cannot be degraded by AROM3 [40]. The PCA titer was increased by about 8 mM to a final titer of 10.3 ± 0.6 mM after 144 h cultivation upon *dapC* gene deletion (Fig. 2C). The accumulated PCA is not expected to have toxic effects*,* as growth of *C.* *glutamicum* was demonstrated for concentrations up to 500 mM PCA [41], but this by-product formation may decrease tyrosol production and is aimed to be reduced.

In *C.* *glutamicum*, PCA is synthesized from the precursor of shikimate, 3-dehydroshikimate, by 3-dehydroshikimate dehydratase encoded by *qsuB* (Fig. 2A). The *qsuB* gene is part of an operon with *qsuA*, *qsuC* and *qsuD*. The shikimate dehydrogenase encoded by *qsuD* further acts counterproductive to tyrosol synthesis, as it converts shikimate back to its precursor dehydroshikimate. Meanwhile, the expression of *qsuC*, encoding 3-dehydroquinate dehydratase, supports the flux towards shikimate [42]. The transcription of the *qsuABCD* operon is activated by QsuR in a chorismate-dependent manner [43]. It was shown that deleting *qsuABD* while enhancing the expression of *qsuC* (Δ*qsuABCD*::P_*tuf*_-*qsuC*) increased the flux from 3-dehydroquinate towards shikimate [44], thereby benefiting the production of shikimate-derived metabolites. We, therefore, performed the chromosomal exchange (Δ*qsuABCD*::P_*tuf*_-*qsuC,* abbreviated here to Δ*qsuABD*) and measured the production of aromatic compounds by the resulting strain AROM3 Δ*dapC* Δ*qsuABD* (*tyrA*_*Ec*_^fbr^) (*ARO10*_*Sc*_). The gene deletion of *qsuABD* successfully reduced PCA formation to less than 0.3 mM (Fig. 2D), but tyrosol accumulation did not increase. Supplementation of l-tyrosine and l-lysine did not increase final tyrosol titers (Fig. S4D). Thus, while tyrosol production without concomitant PCA and l-tyrosine accumulation was achieved (a benefit for downstream processing), the carbon flux could not fully be rerouted to tyrosol production.

### Native synthesis of 4-OH-phenylpyruvate via transamination

We expected that the bifunctional chorismate mutase/prephenate dehydrogenase from *E. coli* encoded by *tyrA*_*Ec*_^fbr^ was necessary to synthesize 4-OH-phenylpyruvate as a precursor for tyrosol synthesis via Aro10_*Sc*_. However, the observation that tyrosol production decreased together with the l-tyrosine synthesis upon deletion of *dapC* prompted us to consider the involvement of l-tyrosine in the tyrosol production pathway. If l-tyrosine were a precursor of 4-OH-phenylpyruvate, an involvement of TyrA_*Ec*_^fbr^ would not be required. Therefore, we compared tyrosol production of AROM3 (*ARO10*_*Sc*_)(*tyrA*_*Ec*_^fbr^) to the respective empty vector carrying strain AROM3 (*ARO10*_*Sc*_)(EV1).

**Fig. 3:**
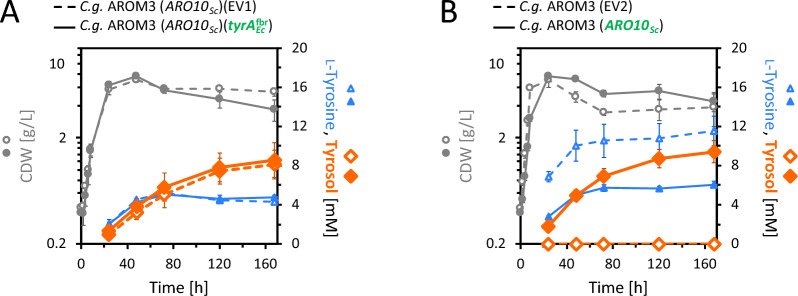
Tyrosol production via expression of *ARO10*_*Sc*_ in AROM3. Growth (CDW) and production of l-tyrosine and tyrosol for *C.* *glutamicum* AROM3 (*ARO10*_*Sc*_)(*tyrA*_*Ec*_^fbr^) compared to the respective empty vector control strain (**A**) (glucose was depleted after 120 h). *C.* *glutamicum* AROM3 with the single plasmid pECXT-P_syn_-*ARO10*_*Sc*_ (which exhausted the glucose after 72 h) is compared to its empty vector control strain (which depleted the glucose after 48 h) (**B**). Values and error bars represent means and standard deviations of triplicate shake flask cultivations

Surprisingly, the strain expressing only *ARO10*_*Sc*_ produced as much tyrosol as the strain expressing *ARO10*_*Sc*_ in combination with *tyrA*_*Ec*_^fbr^ (Fig. 3A), indicating that *tyrA*_*Ec*_^fbr^ was not required for tyrosol production. Tyrosol production was thus enabled solely by expression of *ARO10*_*Sc*_. In a follow-up experiment, AROM3 (*ARO10*_*Sc*_) without a second plasmid produced 9.4 ± 1.1 mM (1.30 ± 0.15 g/L) (Fig. 3B), while no tyrosol was detected for the respective control strain AROM3 (EV2). This corroborated that *ARO10*_*Sc*_ is required and sufficient to enable tyrosol production by AROM3.

**Fig. 4:**
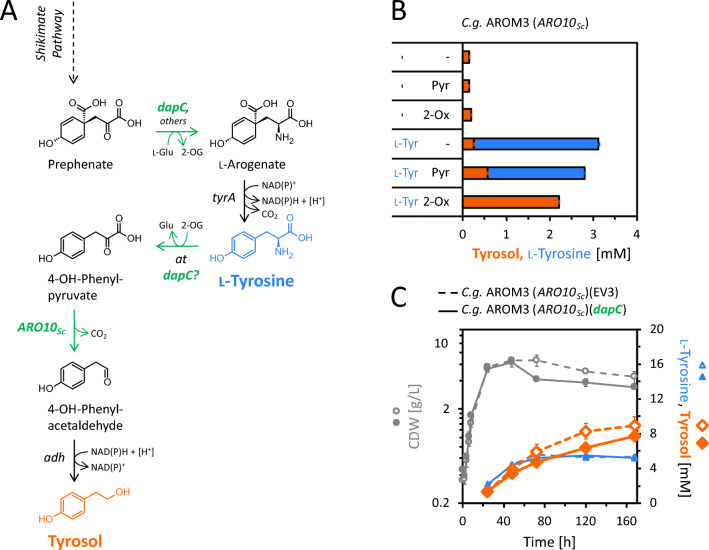
Tyrosol production with native 4-OH-phenylpyruvate synthesis by strain AROM3 (*ARO10*_*Sc*_). **A** Tyrosol synthesis pathway with 4-OH-phenylpyruvate as intermediate, synthesized via prephenate dehydrogenase from *E. coli* (heterologously expressed by *tyrA*_*Ec*_^fbr^) or via native aminotransferases (encoded by undefined genes *at* and putative *dapC*) from l-tyrosine. **B** Conversion of l-tyrosine to tyrosol by *C.* *glutamicum* AROM3 (*ARO10*_*Sc*_) in a whole-cell enzyme assay with permeabilized cells upon supplementation of 3 mM l-tyrosine (l-Tyr) and surplus addition of amino acceptors pyruvate (Pyr) or 2-oxoglutarate (2-OG), as indicated. **C** Influence of plasmid-based overexpression of *dapC* on growth (CDW) and production of l-tyrosine and tyrosol by strain *C.* *glutamicum* AROM3 (*ARO10*_*Sc*_). Glucose was depleted after 72 h by the empty vector strain AROM3 (*ARO10*_*Sc*_)(EV3) and after 120 h by AROM3 (*ARO10*_*Sc*_)(*dapC*). Values and error bars represent means and standard deviations of triplicate cultivations

Following this unexpected observation, the metabolic source of the precursor of tyrosol production in *C.* *glutamicum* AROM3 (*ARO10*_*Sc*_) was sought. As expression of the prephenate dehydrogenase gene from *E. coli* was not required and *C.* *glutamicum* lacks a prephenate dehydrogenase, conversion of l-tyrosine to 4-OH-phenylpyruvate by transamination was regarded (Fig. 4A). To test if AROM3 (*ARO10*_*Sc*_) is able to produce tyrosol from l-tyrosine, we measured conversion of externally added l-tyrosine (3 mM) by permeabilized cells. Transamination of l-tyrosine requires a 2-oxo carboxylic acid, e.g., 2-oxoglutarate, as amine acceptor. Therefore, the test was conducted with and without additional supplementation of the amine acceptors pyruvate or 2-oxoglutarate (Fig. 4B). Tyrosol concentrations with supplementation of either pyruvate, 2-oxoglutarate or l-tyrosine alone hardly differed from the negative control without supplements (all < 0.3 mM). When l-tyrosine was supplemented in addition to pyruvate, 0.6 mM tyrosol accumulated, and 2.2 mM l-tyrosine remained. In contrast, the combined addition of 2-oxoglutarate and l-tyrosine resulted in a titer of 2.2 mM tyrosol with no detectable residual l-tyrosine. As expected for 2-oxoglutarate-dependent aminotransferase reactions, additional l-glutamate was formed in this set up (about + 3 mM). Taken together, native aminotransferases of *C.* *glutamicum* depending on 2-oxoglutarate (and to a lesser extent pyruvate) appear to accept l-tyrosine for transamination to 4-OH-phenylpyruvate, which subsequently is converted to tyrosol.

As the addition of 2-oxoglutarate and 3 mM l-tyrosine cocomitantly increased l-glutamate accumulation by 3 mM, we inferred that 3 mM 4-OH-phenylpyruvate were formed, but observed that only 2.2 mM tyrosol was produced. Thus, since neither 4-OH-phenylpyruvate nor 4-OH-phenylacetaldehyde were detected, about 1/3 mol/mol of 4-OH-phenylpyruvate did not contribute to tyrosol formation, but was lost. To test whether 4-OH-phenylpyruvate is unstable, 5 mM 4-OH-phenylpyruvate were incubated in sterile CGXII minimal medium under standard cultivation conditions (30 °C, 120 rpm). Almost no 4-OH-phenylpyruvate was detectable after 24 h, but instead mainly 4-formylphenol was formed among other compounds (Figure S5).

As a cross-check, we investigated growth of the l-arogenate dehydrogenase deficient deletion mutant *C.* *glutamicum* Δ*tyrA.* In contrast to *E. coli*, the *tyrA* gene from *C.* *glutamicum* encodes a dehydrogenase that is specific for l-arogenate but does not accept prephenate [35], a trait shared among *Actinobacteria* [45]. As anticipated, this strain showed l-tyrosine auxotrophy (Figure S6). Transforming this strain with pVWEx4-*tyrA*_*Ec*_^fbr^ for expression of the bifunctional chorismate mutase/prephenate dehydrogenase from *E. coli* restored growth in minimal medium (Figure S6).This indicates that, for one, *C.* *glutamicum* does not possess a prephenate dehydrogenase itself and, secondly, that 4-OH-phenylpyruvate produced by *E. coli*’s TyrA is converted to l-tyrosine by the native aminotransferases of *C.* *glutamicum.*

In l-lysine biosynthesis, DapC accepts *N*-succinyl-2-amino-6-oxopimelate as 2-oxo acid substrate and genetic evidence indicated it is one of the aminotransferases converting the 2-oxo acid prephenate to l-arogenate [29]. However, it is unknown whether DapC may also convert l-tyrosine to the 2-oxo acid 4-OH-phenylpyruvate. To test if *dapC* overexpression provides more 4-OH-phenylpyruvate from l-tyrosine as substrate for Aro10_*Sc*_ (Fig. 4A), we constructed strain AROM3 (*ARO10*_*Sc*_)(*dapC*). However, the overexpression of *dapC* did not increase tyrosol production as compared to the parental strain (Fig. 4C).

### Regarding the last step in tyrosol synthesis: potential alcohol dehydrogenases

**Fig. 5:**
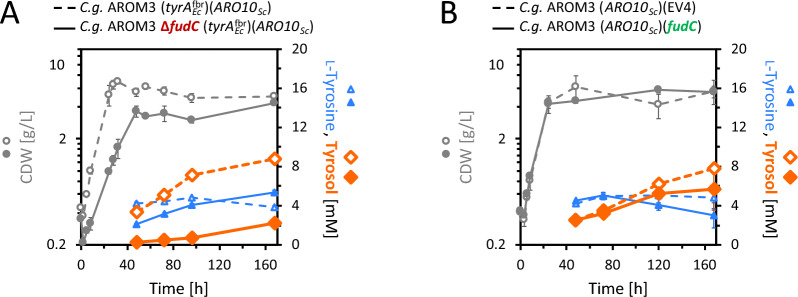
Involvement of the furfural dehydrogenase FudC in tyrosol synthesis. Growth (CDW) and production of l-tyrosine and tyrosol by *C.* *glutamicum* AROM3 (*tyrA*_*Ec*_^fbr^)(*ARO10*_*Sc*_) (which depleted all glucose after 96 h) compared to its respective *fudC*-deletion strain (here, glucose was depleted after 168 h) (**A**) and comparison of *fudC* overexpression in AROM3 (*ARO10*_*Sc*_)(*fudC*) to its respective empty vector control strain (**B**). Both strains had consumed all glucose after 120 h. Values and error bars represent means and standard deviations of triplicate shake flask cultivations

It is not known which native alcohol dehydrogenase(s) reduce(s) 4-OH-phenylacetaldehyde to tyrosol in *C. glutamicum*. Studies conducted in *E. coli* showed that tyrosol production benefits from overexpressing 4-OH-phenylacetaldehyde-accepting alcohol dehydrogenases, such as YahK from *E. coli* [46] or ADH6 from *S. cerevisiae* [39]. We conducted a protein–protein BLAST search [47] for the respective protein sequences (Yahk_*Ec*_ UniProt: P75691; ADH6_*Sc*_ UniProt: Q04894) against the proteins encoded in the genome of *C.* *glutamicum* (Table S1). The search revealed that the protein with the highest amino acid sequence similarity to both alcohol dehydrogenases (with 46% identity to YahK and 37.5% identity to ADH6) is the furfural dehydrogenase FudC, encoded by cg0400 (also known as NCgl0324 or Cgl0331). FudC does not only reduce the heterocyclic aromatic aldehyde furfural [48], but also showed reductase activity with aromatic benzaldehydes in vivo, such as 4-formylphenol, protocatechuic aldehyde, and vanillin [49]. To assess the involvement of FudC in tyrosol synthesis, we deleted *fudC* in AROM3 and compared tyrosol production. Indeed, the synthesis of tyrosol was reduced by 75% upon deletion of the *fudC* gene (Fig. 5A). Moreover, 4-OH-phenylacetaldehyde was detected by GC–MS analysis exclusively in the supernatants of the *fudC* deletion strain. The residual production of tyrosol (2.2 ± 0.3 mM) by AROM3 Δ*fudC* indicated that at least one further alcohol dehydrogenase is involved in the reduction of 4-OH-phenylacetaldehyde to tyrosol. Apparently, the substrate scope of FudC is not limited to furan-ring aldehydes and benzaldehydes, but also encompasses phenylacetaldehydes.

Next, we tested overexpression of *fudC* in AROM3 (*ARO10*_*Sc*_), but this approach did not enhance tyrosol synthesis (Fig. 5B). In line with the finding that 4-OH-phenylacetaldehyde is absent from supernatants with intact *fudC*, the reduction of 4-OH-phenylacetaldehyde appeared not to be a rate-limiting step.

### Production of tyrosol via tyramine

**Fig. 6:**
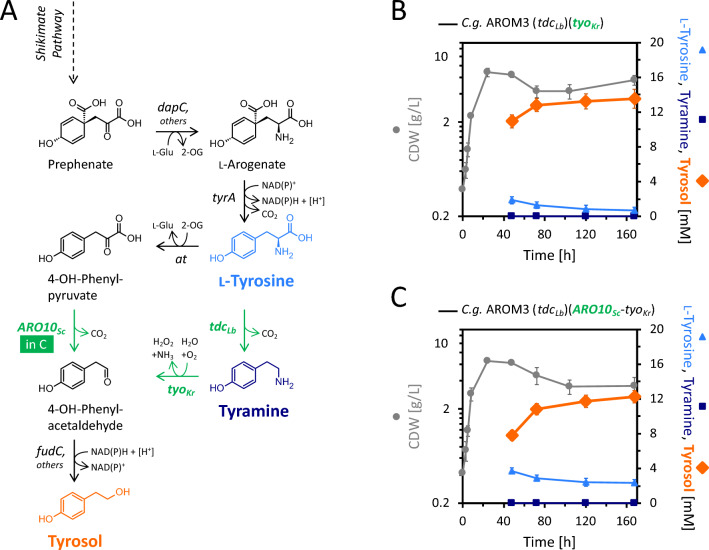
Tyrosol production via plasmid-based expression of *tyo*_*Kr*_ in strain AROM3 (*tdc*_*Lb*_) and combination with *ARO10*_*Sc*_. **A** Tyrosol synthesis pathway with tyramine as intermediate, including the overexpression of the heterologous genes *tdc*_*Lb*_ and *tyo*_*Kr*_ (green), encoding l-tyrosine decarboxylase from *L. brevis* and tyramine oxidase from *K. rhizophila*, respectively, overexpression of *ARO10*_*Sc*_ and the involvement of the furfural dehydrogenase encoded by *fudC* (among other alcohol dehydrogenases). **B**, **C** Comparison of growth (CDW) and production of l-tyrosine, tyramine, and tyrosol for *C.* *glutamicum* AROM3 (*tdc*_*Lb*_)(*tyo*_*Kr*_) (**B**) (glucose was exhausted after 48 h) and for the strain with additional overexpression of *ARO10*_*Sc*_ (**C**) (glucose was depleted after 72 h). Values and error bars represent means and standard deviations of triplicate shake flask cultivations

During the synthesis of tyrosol from l-tyrosine via 4-OH-phenylpyruvate as intermediate, 1/3 mol/mol is lost presumably due to the instability of 4-OH-phenylpyruvate. However, there is an alternative pathway from l-tyrosine towards 4-OH-phenylacetaldehyde using an interchanged order of deamination and decarboxylation. Thereby, tyramine is formed as an intermediate instead of the unstable 4-OH-phenylpyruvate in the *ARO10*_*Sc*_ pathway. Previously, we demonstrated that tyramine is stable under cultivation conditions and is not degraded by AROM3 [32]. To this end, we used a tyramine producing strain obtained by metabolic engineering of *C.* *glutamicum* AROM3 expressing the l-tyrosine decarboxylase gene from *Levilactobacillus brevis* (*tdc*_*Lb*_) [32]. Tyramine can be oxidized to 4-OH-phenylacetaldehyde, e.g., by the tyramine oxidase Tyo from *Micrococcus luteus* (EC 1.4.3.4), as shown for tyrosol production by *E. coli* [15]. Subsequently, 4-OH-phenylacetaldehyde is reduced to tyrosol by an alcohol dehydrogenase. In this study, we used a homolog of the tyramine oxidase from *Kocuria rhizophila* (with 93% amino acid sequence identity to Tyo_*Ml*_) for the first step and opted on native alcohol dehydrogenases (Adh), including FudC for the second step (Fig. 6A). Upon cultivation of the constructed strain AROM3 (*tdc*_*Lb*_)(*tyo*_*Kr*_), 13.5 ± 1.1 mM tyrosol (equivalent to 1.87 ± 0.15 g/L, Fig. 6B) was produced, while no tyrosol was detected for the respective empty vector control AROM3 (*tdc*_*Lb*_)(EV1) (Figure S7). Besides tyrosol, also l-tyrosine was present in the supernatants, but tyramine was not detected. Concluding, the expression of *tyo*_*Kr*_ in the tyramine overproducing strain is a successful strategy to produce tyrosol, yet the decarboxylation of l-tyrosine to tyramine by Tdc_*Lb*_ seems to be the bottleneck of tyrosol production by this strain.

As for both strains, AROM3 (*ARO10*_*Sc*_) and AROM3 (*tdc*_*Lb*_)(*tyo*_*Kr*_), the intermediate l-tyrosine accumulated, the combination of both pathways was sought. Therefore, the codon harmonized *ARO10*_*Sc*_ gene was cloned into the plasmid pVWEx4-*tyo*_*Kr*_ and the constructed strain AROM3 (*tdc*_*Lb*_)(*ARO10*_*Sc*_-*tyo*_*Kr*_) harbored both pathways. However, this strain produced less tyrosol and more l-tyrosine remained as compared to AROM3 (*tdc*_*Lb*_)(*tyo*_*Kr*_) (Fig. 6C).

**Fig. 7:**
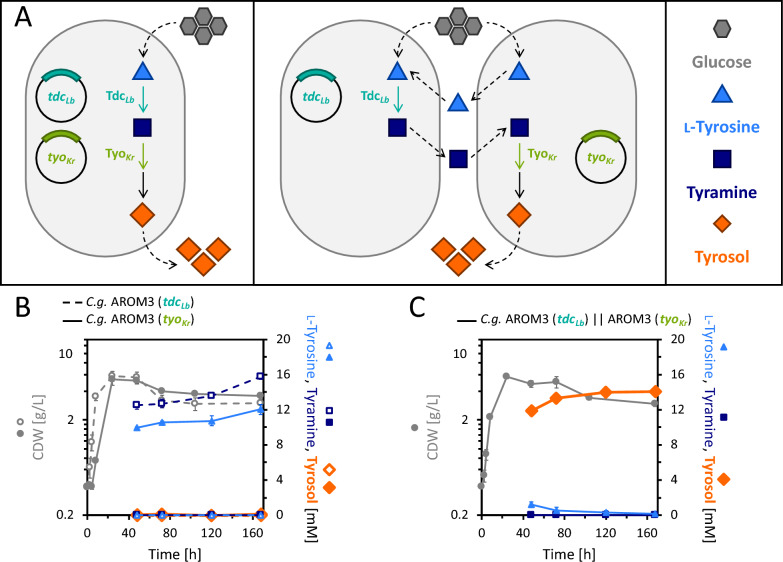
Division of labor for tyrosol production via co-cultivation of AROM3 strains expressing either *tdc*_*Lb*_ or *tyo*_*Kr*_. **A** Principle of tyrosol production by AROM3 carrying two plasmids (left) against labor division with single plasmid carrying AROM3 strains (right). **B**, **C** Comparison of growth (CDW) and production of l-tyrosine, tyramine, and tyrosol for AROM3 (*tdc*_*Lb*_) and AROM3 (*tyo*_*Kr*_) in monoculture each (**B**) and both strains in co-culture with 50%-CDW inoculation (**C**). Glucose was depleted after 48 h for all cultivations. Values and error bars represent means and standard deviations of triplicate shake flask cultivations

As the single plasmid carrying strain AROM3 (*tdc*_*Lb*_) decarboxylated all produced l-tyrosine to tyramine (Fig. 7B), reduced expression of *tdc*_*Lb*_ in AROM3 (*tdc*_*Lb*_)(*tyo*_*Kr*_) as cause of the second plasmid was considered. Since division of labor is a powerful tool to reduce metabolic burden [50], AROM3 strains carrying the single plasmids with either *tdc*_*Lb*_ or *tyo*_*Kr*_ were grown in mono- or co-culture (with 50% initial CDW of both strains) (Fig. 7). This approach revealed an increase in tyrosol titer to 14.1 ± 0.3 mM (1.95 ± 0.04 g/L) and a decrease in remaining l-tyrosine to less than 0.2 mM. The results indicate that decarboxylation of l-tyrosine by plasmid-based expression of the tyramine decarboxylase gene *tdc*_*Lb*_ is more effective in the single plasmid carrying strain, overall demonstrating the potential of this measure.

All in all, the highest production titers of about 14 mM tyrosol were achieved via the tyramine pathway by expression of *tdc*_*Lb*_ and *tyo*_*Kr*_, thus clearly outperforming the pathway via 4-OH-phenylpyruvate applied in AROM3 (*ARO10*_*Sc*_). Not only the final titer was increased by 44%, but the production was also faster, leading to a more than doubled maximal volumetric productivity (Table 1). In addition, l-tyrosine concentrations have been substantially diminished, thereby facilitating downstream processing.

**Table 1:** Comparison of production parameters regarding the two pathways with either expression of *ARO10*_*Sc*_ or *tdc*_*Lb*_ and *tyo*_*Kr*_

Strains	Titer [mM]	Titer [g/L]	Yield on glucose [g/g]	Max. volumetric productivity [g/L/h]	Remaining byproducts	
					Tyrosine [mM]	PCA [mM]
AROM3 (*ARO10*_*Sc*_)	9.4 ± 1.1	1.30 ± 0.15	0.033	0.014	6.0 ± 0.3	3.5 ± 0.4
AROM3 (*tdc*_*Lb*_)(*tyo*_*Kr*_)	13.5 ± 1.1	1.87 ± 0.15	0.047	0.032	0.7 ± 0.4	3.4 ± 0.3
AROM3 (*tdc*_*Lb*_) + AROM3 (*tyo*_*Kr*_)	14.1 ± 0.3	1.95 ± 0.04	0.049	0.034	0.2 ± 0.2	2.1 ± 0.1

## Discussion

In this study, tyrosol production via expression of the gene *ARO10*_*Sc*_ encoding the phenylpyruvate decarboxylase was shown to proceed via synthesis of 4-OH-phenylpyruvate from l-tyrosine by native aminotransferases in *C.* *glutamicum.* This pathway was more efficient for tyrosol production than the pathway involving the heterologous prephenate dehydrogenase TyrA_*Ec*_^fbr^ from *E. coli*. As 4-OH-phenylpyruvate is not part of the native l-tyrosine synthesis pathway of *C.* *glutamicum,* which lacks prephenate dehydrogenase activity [35], the transamination of 4-OH-phenylpyruvate and l-tyrosine does not seem to be beneficial for growth of *C.* *glutamicum,* but may be relevant for degrading l-tyrosine. Interestingly, *C.* *glutamicum* has been described to utilize l-tyrosine as sole nitrogen source, but not as sole carbon source [51]; however, the mechanism remained unclear. Here, we have shown that l-tyrosine was not converted as sole carbon source unless 2-oxoglutarate was added and formation of l-glutamate was demonstrated (Fig. 4B). Aminotransferases generally show a broad substrate spectrum. The branched-chain aminotransferase IlvE and the aromatic aminotransferase AroT accept 4-OH-phenylpyruvate as substrate and l-glutamate as amino donor, albeit with lower activities (namely, 8.8 and 2.4 µmol/min/mg_protein_, respectively) compared to phenylpyruvate (13.6 and 10.7 µmol/min/mg_protein_, respectively) [52]. Although l-glutamate is the preferred amino donor of AroT, the enzyme exhibits 60% of its activity also with the amino donor l-alanine. In our experiments, only 20% of the l-tyrosine was converted with addition of pyruvate as amino acceptor, indicating the (additional) involvement of aminotransferases other than AroT. Genetic evidence revealed that aminotransferase DapC (part of l-lysine biosynthesis in *C.* *glutamicum*) is active with prephenate and l-arogenate [29]. A BLAST search with yeast aminotransferase Aro8_*Sc*_, which is known to catalyze the transamination of 4-OH-phenylpyruvate to l-tyrosine [53], against the proteins encoded by the *C.* *glutamicum* genome showed the highest score to an uncharacterized aminotransferase encoded by cg0931 (NCgl0780). Thus, transamination of l-tyrosine to 4-OH-phenylpyruvate likely involves several aminotransferases in *C.* *glutamicum*.

Considering that supplemented l-tyrosine was converted to tyrosol, the strain demonstrated to be suitable for whole-cell biotransformation, a commonly used approach for the synthesis of bioactive compounds. In *E. coli*, three aminotransferases, TyrB, AspC, and IlvE, catalyze the transamination of 4-OH-phenylpyruvate to l-tyrosine as part of its biosynthesis pathway [54]. *E. coli* overexpressing *ARO10*_*Sc*_ showed a yield on l-tyrosine of 0.45 mol/mol [23]. Notably, with a yield on l-tyrosine of 0.67 mol/mol (Fig. 4B), the here tested *C.* *glutamicum* strain overexpressing *ARO10*_*Sc*_ outcompeted *E. coli.* Additional overexpression of the aminotransferase gene *ARO8*_*Sc*_ improved yields on l-tyrosine to 0.87 mol/mol in *E. coli* [23], which may improve *C.* *glutamicum*-based biotransformation of l-tyrosine to tyrosol, as well.

Compared to whole-cell biotransformation, the de novo production of tyrosol is a more feasible approach due to the utilization of simple carbon and nitrogen sources (glucose and ammonia), instead of l-tyrosine and 2-oxoglutarate. The g/L scale fermentative production by AROM3 (*ARO10*_*Sc*_) shown here may be further improved regarding the transamination reaction, since some l-tyrosine remained at the end of cultivation. Overexpression of the responsible aminotransferase genes might help attaining the thermodynamic equilibrium of the reversible reaction faster; however, it would not alter the equilibrium itself [55]. To improve conversion towards 4-OH-phenylpyruvate, the concentrations of substrates need to be increased or that of the products decreased. The aminotransferase reaction was not hindered by accumulation of its product, 4-OH-phenylpyruvate, which was not detectable (< 0.1 mM) at any measurement timepoint. On the substrate side, however, increasing the intracellular concentration of l-tyrosine by transport engineering might be suitable to push l-tyrosine deamination. To increase the reuptake of excreted l-tyrosine, the genes encoding l-tyrosine import systems, namely, aromatic amino acid importer AroP [56] and/or the l-tyrosine permease TyrP [57]*,* might be overexpressed. Regarding the co-substrate 2-oxoglutarate and the co-product l-glutamate, *E. coli* or *S. cerevisiae* synthesize significantly more 2-oxoglutarate than l-glutamate, whereas *C.* *glutamicum* is known for its high l-glutamate to 2-oxoglutarate ratio [58]. This is beneficial for l-tyrosine synthesis, but not for its deamination to 4-OH-phenylpyruvate. To change this ratio, the reductive amination of 2-oxoglutarate to l-glutamate can be decreased by deleting the l-glutamate dehydrogenase (Gdh) gene. The resulting strain would have a growth benefit, if it regained l-glutamate in the transamination reaction with l-tyrosine. The benefit of *gdh* deletion to enforce transamination reactions has been demonstrated for the production of glutarate [59].

Tyrosol production via Aro10_*Sc*_ may suffer from the instability of the intermediate 4-OH-phenylpyruvate. The observed chemical conversion of 4-OH-phenylpyruvate to 4-formylphenol among other compounds under sterile conditions (Figure S5) is consistent with the observation reported by Doy [60] that the 2-oxo acid 4-OH-phenylpyruvate spontaneously converts in several steps to the aldehyde 4-formylphenol. This degradation product is known to be catabolized by *C.* *glutamicum* [49, 61]. Spontaneous degradation of 4-OH-phenylpyruvate and subsequent catabolic conversion may explain that in the whole-cell biotransformation experiment with AROM3 (*ARO10*_*Sc*_) about 1/3 mol/mol was lost during synthesis of tyrosol from l-tyrosine. Besides chemical instability, enzymatic degradation of 4-OH-phenylpyruvate may be possible, but has not yet been described for *C.* *glutamicum.* However, different phenylpropanoids, such as the structurally similar compound 4-OH-phenylpropionic acid, are degraded by *C.* *glutamicum* via enzymes encoded in the *phd* cluster [62]. Nevertheless, chemical instability of 4-OH-phenylpyruvate necessitates its fast decarboxylation at low substrate concentrations. In this respect, phenylpyruvate decarboxylase Aro10_*Sc*_ that has a lower K_M_ value for indole-3-pyruvate (0.03 mM) than for 4-OH-phenylpyruvate (0.09 mM) [38] was improved by single amino acid exchanges (Aro10_*Sc*_^Q448W^, [38]; Aro10_*Sc*_^D331C^, [63]). Enzyme engineering of Aro10_*Sc*_ may increase its affinity to 4-OH-phenylpyruvate and thus help to overcome instability issues.

The parallel approach of tyrosol production involves the stable intermediate tyramine instead of 4-OH-phenylpyruvate via, first, decarboxylation of l-tyrosine by Tdc_*Lb*_ and, second, deamination to 4-OH-acetaldehyde by Tyo_*Kr*_. Like in the Aro10-utilizing pathway, the irreversible decarboxylation with release of CO_2_ is a strong metabolic driving force [64]. The metabolic pull effect by Tdc explains why about 43 mol-% more tyramine was produced than l-tyrosine by the empty vector control strain [32]. Contrary to the Aro10 pathway, which initially encompasses a reversible transamination, the tyramine oxidase Tyo may exert a second beneficial pull with release of ammonia. The combination of the aforementioned factors; the pull by deamination rather than reversible transamination and the stability of the intermediate tyramine in contrast to 4-OH-phenylpyruvate, may explain the higher production titers that were achieved.

Potential disadvantages of the Tdc–Tyo pathway in comparison with the Aro10 pathway is the metabolic cost of l-glutamate, which might impose a challenge for low l-glutamate producing organisms, such as *E. coli* and *S. cerevisiae*, but less so for *C.* *glutamicum* [58]. Furthermore, the oxidative deamination catalyzed by Tyo generates hydrogen peroxide (H_2_O_2_) [65]. Given that *E. coli* is severely damaged by intracellular H_2_O_2_ concentrations of as little as 1 µM [66], the tyrosol synthesis route via Tyo might present a significant challenge to this bacterium*.* By contrast, *C.* *glutamicum* is known for its resistance towards externally added H_2_O_2_, which is about 10 times higher than that of *E. coli* [67]. The catalase of *C.* *glutamicum* is even marketed because of its high effectiveness (CAS Number 9001–05-2). Concluding, *C.* *glutamicum’*s distinctive characteristics, including high l-glutamate levels and high H_2_O_2_ resistance, favor the Tdc–Tyo pathway. Moreover, as *C.* *glutamicum* synthesizes l-tyrosine via l-arogenate in place of 4-OH-phenylpyruvate, unlike *E. coli* or *S. cerevisiae,* the here shown de novo production of tyrosol is unique due to its exclusion of 4-OH-phenylpyruvate as intermediate.

To reduce the metabolic burden on AROM3 (*tdc*_*Lb*_)(*tyo*_*Kr*_), division of labor was successfully demonstrated for tyrosol production. In contrast to inter-species consortia, for which incompatible growth requirements are common limitations [68], the consortium implemented in this study benefited from the fact that both strains belong to the same species and differ only by the last reaction step of product formation. The uniform requirements for medium and cultivation conditions, as well as similar growth behavior reduce the risk of one strain outgrowing the other, making engineered interdependencies of the strains superfluous [50]. Nevertheless, long-term stability of the co-cultivation process may be ensured through engineering interdependencies or different substrate utilization abilities, e.g., for xylose by one and arabinose by the other strain. This approach would enable the control of the strain ratio by modulation of the substrate supply [50]. As the accumulation of the intermediates l-tyrosine and tyramine was reduced compared to the mono-culture of AROM3 (*tdc*_*Lb*_)(*tyo*_*Kr*_), this suggests *C. glutamicum* efficiently transports l-tyrosine, tyramine, and tyrosol.

Taken together, the expression of the genes *tdc*_*Lb*_ and *tyo*_*Kr*_ in the engineered l-tyrosine producer *C. glutamicum* AROM3 for de novo production of tyrosol is very effective, especially when implemented as a co-culture with single heterologous gene expression.

## Conclusion

We established de novo production of tyrosol from simple carbon and nitrogen sources via two alternative routes. Investigating tyrosol production by heterologous expression of *ARO10*_*Sc*_, which encodes a phenylpyruvate decarboxylase, we demonstrated the involvement of native aminotransferases in the synthesis of 4-OH-phenylpyruvate from l-tyrosine. This finding contributes to a new understanding of l-tyrosine degradation and opens new considerations for the production of l-tyrosine and its derived products. Nevertheless, due to the equilibrium of the transamination reaction and instability of the intermediate 4-OH-phenylpyruvate, we discovered that the alternative route utilizing tyramine as intermediate was superior. The production of tyrosol via heterologous expression of l-tyrosine decarboxylase and tyramine oxidase genes *tdc*_*Lb*_ and *tyo*_*Kr*_ finally resulted in titers of about 14 mM (2 g/L).

## Materials and methods

### Bacterial strains and cultivation conditions

Bacterial strains and plasmids used in this study are listed in Tables 2 and 3, respectively. *E. coli* DH5α was used as a host for plasmid construction and was grown in lysogeny broth (LB) [69] medium in 100 mL unbaffled flasks at 180 rpm at 37 °C. For better comparability of the different strains, overnight cultures of *C.* *glutamicum* were prepared with LB medium supplemented with 10 g/L glucose, to increase biomass formation especially for slowly growing strains, and cultivated at 120 rpm and 30 °C. For growth and production experiments, overnight cultures were harvested and used for inoculation of CGXII minimal medium [70], with 40 g/L glucose to an optical density at 600 nm (OD_600_) of 1. The cultivation was performed in 100 ml baffled flasks with a filling volume of 10 vol-% at 120 rpm and 30 °C in technical triplicates, if not stated otherwise. Antibiotics (25 mg/L kanamycin, and 5 mg/L tetracycline) as well as 1 mM isopropyl-β-d−1-thiogalactopyranoside (IPTG) to induce gene expression were supplemented if required. The amino acid l-phenylalanine (0.5 mM) was added for cultivation of all AROM3 derived strains. Growth was monitored by measuring the OD_600_ using a V-1200 spectrophotometer (VWR, Radnor, PA, USA) and biomass concentrations were calculated according to the experimentally determined correlation: cell dry weight concentration (CDW) = 0.347 × OD_600_ [g/L], which was similar to previously published results [71]. Glucose depletion was checked using glucose test strips (Macherey–Nagel GmbH & Co. KG, Düren, Germany).

**Table 2:** Bacterial strains used in this study

Strain	Relevant characteristics	References
*E. coli* DH5α	Δ*lacU169* (*φ80lacZ* Δ*M15*), *supE44*, *hsdR17*, *recA1*, *endA1*, *gyrA96*, *thi-1*,* relA1*	[73]
*C. glutamicum* WT	*C. glutamicum* ATCC 13032	ATCC
AROM3	*C. glutamicum* C1* Δ*ldhA* Δ*vdh*::P_*ilvC*_-*aroG*_*Ec*_^D146N^ *trpE*^TTG^ *pheA*^TTG^	[29]
AROM3 Δ*csm*	AROM3 with inframe deletion of *csm*	This study
AROM3E Δ*csm* (EV1)	AROM3 Δ*csm* carrying pECXT-P*syn* and pVWEx4	This study
AROM3E Δ*csm* (*tyrA*_*Ec*_^fbr^)	AROM3 Δ*csm* carrying pECXT-P*syn* and pVWEx4-*tyrA*_*Ec*_^fbr^	This study
AROM3 (*tyrA*_*Ec*_^fbr^) (*ARO10*_*Sc*_)	AROM3 carrying pVWEx4-*tyrA*_*Ec*_^fbr^ and pECXT-P_syn_-*ARO10*_*Sc*_	This study
AROM3 Δ*dapC*	AROM3 with inframe deletion of *dapC*	This study
AROM3 Δ*dapC* (*tyrA*_*Ec*_^fbr^) (*ARO10*_*Sc*_)	AROM3 Δ*dapC* carrying pVWEx4-*tyrA*_*Ec*_^fbr^ and pECXT-P_syn_-*ARO10*_*Sc*_	This study
AROM3 Δ*dapC* Δ*qsuABD*	AROM3 Δ*dapC* with replacement Δ*qsuABCD*::P_*tuf*_-*qsuC*	This study
AROM3 Δ*dapC* Δ*qsuABD* (*tyrA*_*Ec*_^fbr^) (*ARO10*_*Sc*_)	AROM3 Δ*dapC* Δ*qsuABCD*::P_*tuf*_-*qsuC* carrying pVWEx4-*tyrA*_*Ec*_^fbr^ and pECXT-P_syn_-*ARO10*_*Sc*_	This study
AROM3 (*ARO10*_*Sc*_) (EV1)	AROM3 carrying pECXT-P_syn_-*ARO10*_*Sc*_ and pVWEx4	This study
AROM3 (EV2)	AROM3 carrying pECXT-P*syn*	This study
AROM3 (*ARO10*_*Sc*_)	AROM3 carrying pECXT-P_syn_-*ARO10*_*Sc*_	This study
Δ*tyrA*	*C. glutamicum* WT with inframe deletion of *tyrA*	This study
Δ*tyrA* (*tyrA*_*Ec*_^fbr^)	Δ*tyrA* carrying pVWEx4-*tyrA*_*Ec*_^fbr^	This study
AROM3 (*ARO10*_*Sc*_) (EV3)	AROM3 carrying pECXT-P_syn_-*ARO10*_*Sc*_ and pEKEx2	This study
AROM3 (*ARO10*_*Sc*_) (*dapC*)	AROM3 carrying pECXT-P_syn_-*ARO10*_*Sc*_ and pEKEx2-*dapC*	This study
AROM3 Δ*fudC*	AROM3 with inframe deletion of *fudC*	This study
AROM3 Δ*fudC* (*tyrA*_*Ec*_^fbr^) (*ARO10*_*Sc*_)	AROM3 Δ*fudC* carrying pVWEx4-*tyrA*_*Ec*_^fbr^ and pECXT-P_syn_-*ARO10*_*Sc*_	This study
AROM3 (*ARO10*_*Sc*_) (EV4)	AROM3 carrying pECXT-P_syn_-*ARO10*_*Sc*_ and pVWEx1	This study
AROM3 (*ARO10*_*Sc*_) (*fudC*)	AROM3 carrying pECXT-P_syn_-*ARO10*_*Sc*_ and pVWEx1-*fudC*	This study
AROM3 (*tdc*_*Lb*_) (EV1)	AROM3 carrying pECXT99A-*tdc*_*Lb*_ and pVWEx4	This study
AROM3 (*tdc*_*Lb*_) (*tyo*_*Kr*_)	AROM3 carrying pECXT99A-*tdc*_*Lb*_ and pVWEx4-*tyo*_*Kr*_	This study
AROM3 (*tdc*_*Lb*_) (*ARO10*_*Sc*_-*tyo*_*Kr*_)	AROM3 carrying pECXT99A-*tdc*_*Lb*_ and pVWEx4-*ARO10*_*Sc*_-*tyo*_*Kr*_	This study
AROM3 (*tdc*_*Lb*_)	AROM3 carrying pECXT99A-*tdc*_*Lb*_	[32]
AROM3 (*tyo*_*Kr*_)	AROM3 carrying pVWEx4-*tyo*_*Kr*_	This study

**Table 3:** Plasmids used in this study

Plasmid	Relevant characteristics		References
pECXT-P_syn_	*C. glutamicum*/*E. coli* shuttle vector for constitutive overexpression, P_*syn*_, pGA1 oriV_*Cg*_	Tet^R^	[74]
pECXT-P_syn_-*ARO10*_*Sc*_	Derivative of pECXT-P_syn_ for overexpression of codon harmonized *ARO10* from *S. cerevisiae* with an optimized RBS (gene sequence: Table S2)	Tet^R^	This study
pECXT99A-*tdc*_*Lb*_	Derivative of pECXT99A (*C.* *glutamicum*/*E. coli* shuttle vector for inducible overexpression, P_*trc*_, *lacI*^q^, pGA1 oriV_*Cg*_) for overexpression of codon-harmonized *tdc* from *L. brevis* with an optimized RBS	Tet^R^	[32]
pEKEx2	*C. glutamicum*/*E. coli* shuttle vector for inducible overexpression, P_*tac*_, *lacI*^q^, pBL1 oriV_*Cg*_	Km^R^	[75]
pEKEx2-*dapC*	Derivative of pEKEx2 for overexpression of *dapC* (cg1253) from *C.* *glutamicum*	Km^R^	[29]
pK19-Δ*csm*	pK19*mobsacB* with a construct for the deletion of *csm* (cg0975)	Km^R^	[76]
pK19-Δ*dapC*	pK19*mobsacB* with a construct for the deletion of *dapC* (cg1253)	Km^R^	[29]
pK19-Δ*fudC*	pK19*mobsacB* with a construct for the deletion of *fudC* (cg0400)	Km^R^	[49]
pK19-Δ*qsuABCD*::P_*tuf*_-*qsuC*	pK19*mobsacB* with a construct for replacement of *qsuABCD* (cg0501-cg0504) by *qsuC* (cg0503) with an artificial RBS under control of *C.* *glutamicum* promoter *P*_*tuf*_	Km^R^	[77]
pVWEx1	*C. glutamicum*/*E. coli* shuttle vector for inducible overexpression, P_*tac*_, *lacI*^q^, pHM1519 oriV_*Cg*_	Km^R^	[78]
pVWEx1-*fudC*	Derivative of pVWEx1 for overexpression of *fudC* (cg0400) from *C.* *glutamicum* with an optimized RBS	Km^R^	This study
pVWEx4	pVWEx1 derivative with mutation in *repA* for amino acid exchange Gly-429-Asp	Km^R^	[74]
pVWEx4-*ARO10*_*Sc*_-*tyo*_*Kr*_	Derivative of pVWEx4 for overexpression of codon harmonized *ARO10* from *S. cerevisiae* with an optimized RBS (gene sequence: Table S2) and *tyo* from *K. rhizophila* with an optimized RBS (gene sequence: Table S2)	Km^R^	This study
pVWEx4-*tyo*_*Kr*_	Derivative of pVWEx4 for overexpression of *tyo* from *K. rhizophila* with an optimized RBS (gene sequence: Table S2)	Km^R^	This study
pVWEx4-*tyrA*_*Ec*_^fbr^	Derivative of pVWEx4 for overexpression of *tyrA* from *E. coli* with mutations for amino acid exchanges Met-53-Ile and Ala-354-Val	Km^R^	This study

To test for potential growth impairment by addition of tyrosol, the growth experiment was conducted in 48-well FlowerPlates (m2p-labs, Baesweiler, Germany) with a filling volume of 1 mL per well at 1,100 rpm and 30°C. Automated backscatter measurements by the BioLector microcultivation system (m2p-labs, Baesweiler, Germany) was used to follow the growth. Growth rates were calculated for time periods of 7 h (40 measuring points) and the maximal ones were determined for each cultivation. The lag phase was calculated as the delay, for which the maximal growth rate was not yet reached. Therefore, the initial measured backscatter (as measure for the biomass) was inserted into the equation for exponential growth with the equation parameters determined for the maximal specific growth.

For a biotransformation experiment, washed cells were resuspended in phosphate buffer (pH 7) to a CDW of 25 g/L and permeabilized with Triton-X-100 (0.125 vol-%) [72]. The permeabilized cells were diluted to 10 vol-% in 200 mM phosphate buffer (pH 7) containing biotin, PCA, and trace element with concentrations as in CGXII medium. 40 mM pyruvate or 2-oxoglutarate and 3 mM of l-tyrosine were supplemented as indicated. The mixtures were incubated in 24-well Duetz microcultivation plates (Kuhner Shaker GmbH, Herzogenrath, Germany) for 24 h at 30 °C and 180 rpm.

### Molecular biology methods

Plasmid construction and transformation was performed as we described previously [32]. The *ARO10* gene from *S. cerevisiae* (Genbank: NC_001136.10) was codon-harmonized for *C.* *glutamicum* (gene sequence: Table S2) [79] and chemically synthesized in the plasmid pECXT-P_syn_ by Twist Bioscience (South San Francisco, CA, USA). The *tyrA*_*Ec*_, *tyo*_*Kr*_, and *fudC*_*Cg*_ genes were amplified from genomic DNA of the originating organism. To introduce mutations for amino acid exchanges Met-53-Ile and Ala-354-Val into *tyrA*_*Ec*_ PCRs with respective primers (Table S3) were performed and the three obtained fragments were combined during Gibson cloning. Primers with overhangs were used to introduce an optimized ribosome binding site (RBS), which was calculated for each gene specifically using the SalisLab software [80], and overhangs to the respective vectors to enable cloning via Gibson assembly [81]. Chromosomal gene deletions were performed with two-step homologous recombination [70] using the suicide vector pK19*mobsacB* [82] as described previously [77]. Successful plasmid construction and chromosomal gene deletions were verified by PCR and sequencing using the appropriate primers (Table S3).

### Quantification of aromatics by HPLC

Aromatic compounds were quantified with a high-performance liquid chromatography system (1200 series, Agilent Technologies Deutschland GmbH, Waldbronn, Germany). Samples were taken during growth experiments (and stored at -20 °C), diluted with water and centrifuged (14,000 rpm, 20 min). The supernatants were used for reversed-phase HPLC analysis.

5 µl sample were injected for detection of tyrosol, l-tyrosine, and PCA. The analytes were separated using a reversed phase pre- and main column (LiChrospher 100 RP18 (5 μm), 40 mM × 4 mM and CS-ODS 100 RP18 (5 μm), 125 mM × 4 mM, respectively, CS-Chromatographie Service GmbH, Langerwehe, Germany) and a gradient with (A) 0.1 vol-% formic acid and (B) methanol as a mobile phase at a constant flow rate of 0.8 mL/min. The gradient started with 10 vol-% B for 1 min, increased to 70 vol-% within 9 min, further increased to 100 vol-% B within 2 min and stayed there for another 2 min. Afterwards, the starting conditions of 10 vol-% were reached in 2 min and held for another 4 min. A diode array detector (DAD G1315B, 1200 series, Agilent Technologies) was used to detect tyrosol and l-tyrosine at a wavelength of 280 nm, while PCA was measured at a wavelength of 260 nm.

Tyramine and l-glutamate were measured via derivatization with *ortho*-phthaldialdehyde (OPA) using a reversed phase pre- and main column (LiChrospher 100 RP18 EC-5 μm, 40 mM × 4.6 mM and 125 mM × 4.6 mM, respectively, CS-Chromatographie Service GmbH, Langerwehe, Germany). A fluorescence detector with an excitation wavelength of 230 nm and an emission wavelength of 450 nm was used for detection of the fluorescent adducts due to the derivatization. The mobile phase consisted of (A) 0.25 vol‑% sodium acetate (pH 6.0) and (B) methanol and was applied with a gradient described previously [31].

### Identification of aromatics by mass spectrometry

Samples were analyzed using gas chromatography–mass spectrometry (GC–MS), as described previously [31]. Briefly, ethyl acetate was used for extraction, of which 5 µL was injected at a split ratio of 20:1 for GC–MS analysis using a TraceGC gas chromatograph (Thermo Scientific, Waltham, MA, USA) and ISQ ion trap mass spectrometer (Thermo Scientific, Waltham, MA, USA). A TraceGOLD TG-5MS column (30 m × 0.25 mM, film thickness 0.25 μm, Thermo Scientific, Waltham, MA, USA) was used for separation with helium as carrier gas at a constant flow rate of 0.6 mL/min.

## Supplementary Information

Supplementary Material 1.

## Data Availability

All data generated or analyzed during this study are included in this published article and its supplementary information files.
