# Supplementary material for: Two routes for tyrosol production by metabolic engineering of Corynebacterium glutamicum
Source: Biotechnol Biofuels Bioprod. 2025 Apr 5;18:43. doi: 10.1186/s13068-025-02641-6 (PMC11971909; doi:10.1186/s13068-025-02641-6)
Supplement: Supplementary file 1 — Supplementary Material 1. [file 13068_2025_2641_MOESM1_ESM.pdf]

Supplementary Material to

Two routes for tyrosol production by metabolic engineering of *Corynebacterium glutamicum*

Nora Junker[1+], Sara-Sophie Poethe[1+], Volker F. Wendisch[1]

[1] Genetics of Prokaryotes, Faculty of Biology and Center for Biotechnology (CeBiTec), Bielefeld University, Bielefeld, Germany

[+]These authors contributed equally to this work.

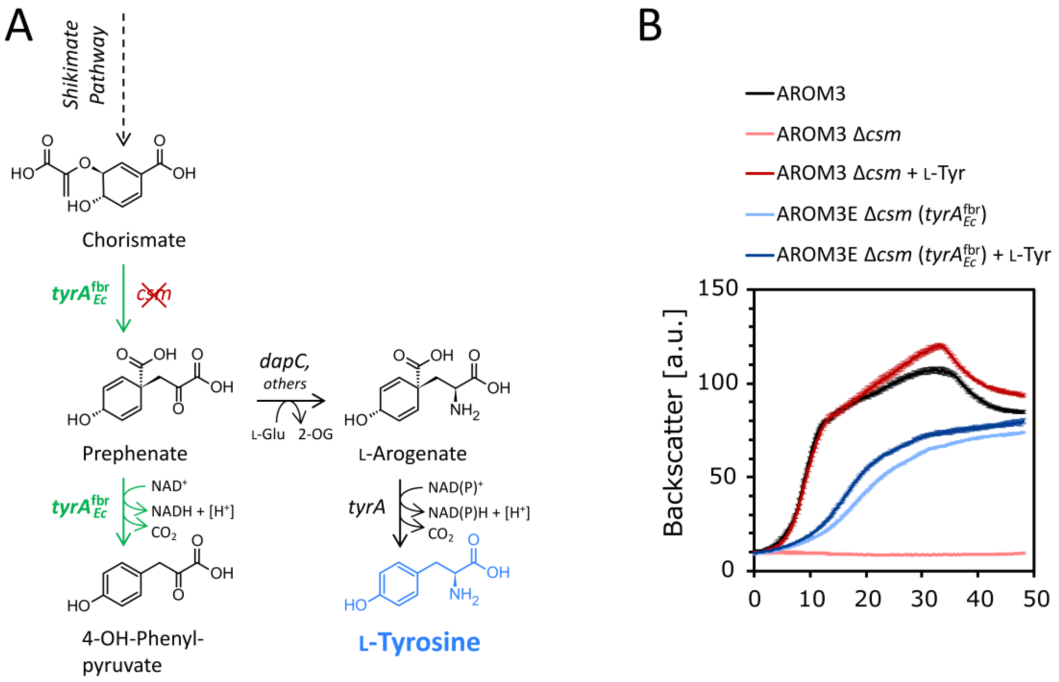

Figure S1: L-Tyrosine auxotrophy of the knockout strain AROM3 Δ*csm* was successfully complemented by expression of *tyrA*$_{Ec}^{fbr}$.

(**A**) L-Tyrosine biosynthesis pathway in *C. glutamicum* with deletion of the chorismate mutase gene *csm* (red) and overexpression of the heterologous gene *tyrA*$_{Ec}^{fbr}$, encoding a feedback resistant mutant of the bifunctional chorismate mutase/prephenate dehydrogenase from *E. coli* (green). Native enzymes (black) are encoded by *dapC: N*-succinyl-aminooxopimelate aminotransferase and *tyrA*: L-arogenate decarboxylase. L-Glu: L-glutamate; 2-OG: 2-oxoglutarate; 4-OH: 4-hydroxy; NAD(P): nicotinamide adenine dinucleotide (phosphate).

(**B**) Growth for *C. glutamicum* AROM3 derived strains with or without supplementation of 1 mM L-tyrosine, cultivated in a BioLector system with automatic measurement of backscatter. Values and error bars represent means and standard deviations of triplicate cultivations.

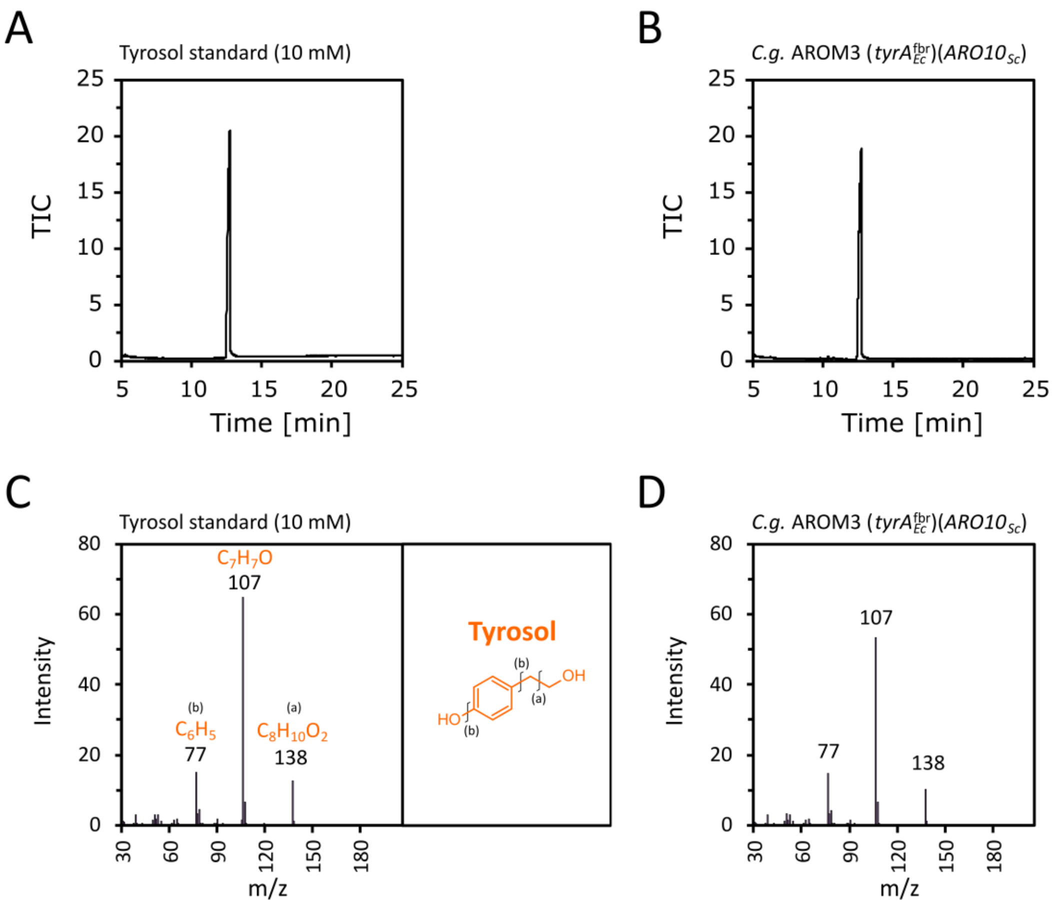

Figure S2: GC-MS analysis of a tyrosol standard and a supernatant sample of *C. glutamicum* AROM3 (*tyrA$_{Ec}$*^{fbr})(*ARO10$_{Sc}$*). For comparison, tyrosol dissolved in water (10 mM) (**A** and **C**) and the supernatant sample (**B** and **D**) were both extracted with ethyl acetate and measured via GC-MS, resulting in the GC chromatogram (**A** and **B**) and the respective mass spectrum corresponding to the peak with the highest abundance (at a retention time of 12.6 min) (**C** and **D**). The mass spectra of the standard and the supernatant sample show a similar pattern of the tyrosol mother ion (138 m/Z) and the characteristic fragment ions (77 m/z and 107 m/z).

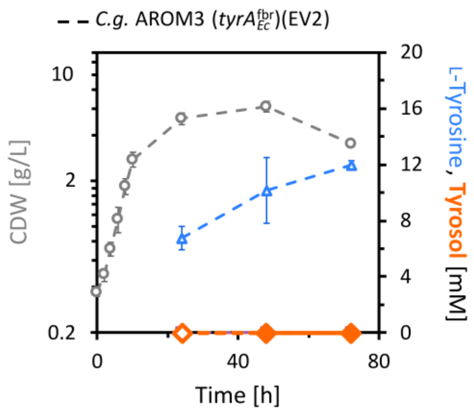

Figure S3: L-Tyrosine production with plasmid-based expression of *tyrA$_{Ec}$*^{fbr} in the empty vector carrying strain *C. glutamicum* AROM3. Values and error bars represent means and standard deviations of triplicate cultivations.

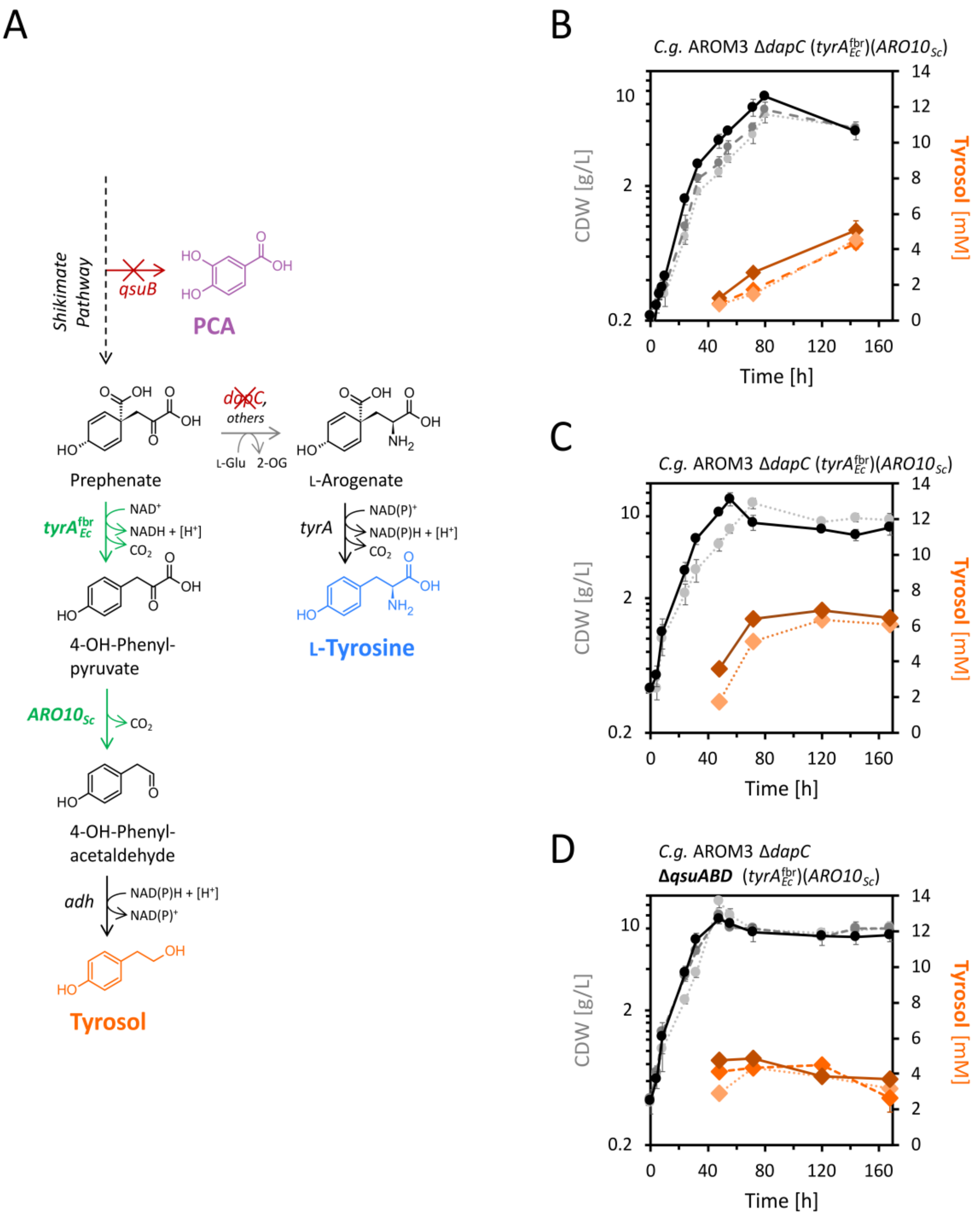

Figure S4: Tyrosol production with *dapC* and *qsuABD* deletion strains.

(A) Tyrosol synthesis pathway with 4-OH-phenylpyruvate as intermediate via the overexpression of the heterologous genes $tyrA_{Ec}^{fbr}$ and $ARO10_{Sc}$ (green) and gene deletions of *qsuB* and *dapC,* encoding 3-dehydroshikimate dehydratase and *N*-succinyl-aminooxopimelate aminotransferase, respectively, are indicated by red crosses. Native enzymes (black) are encoded by *tyrA*: L-arogenate decarboxylase and *adh*: alcohol dehydrogenase. PCA: protocatechuate; Glu: L-glutamate; 2-OG: 2-oxoglutarate; 4-OH: 4-hydroxy; NAD(P): nicotinamide adenine dinucleotide (phosphate).

Growth (CDW) and production of tyrosol are shown for *C. glutamicum* AROM3 Δ*dapC* ($tyrA_{Ec}^{fbr}$)($ARO10_{Sc}$) (**B** and **C**), and for *C. glutamicum* AROM3 Δ*dapC* Δ*qsuABD* ($tyrA_{Ec}^{fbr}$)($ARO10_{Sc}$) (**D**). (**B**) The strain was cultivated in CGXII medium without L-tyrosine or L-lysine supplementation (dotted line), with 0.1 mM L-tyrosine (dashed line) or with 0.5 mM L-tyrosine (full line). (**C**

and **D**) The medium was supplemented with 0.5 mM L-tyrosine (dashed line), with 1 mM L-tyrosine plus 1 mM L-lysine (full line), or without L-tyrosine or 1 mM L-lysine (dotted line).

Values and error bars represent means and standard deviations of triplicate cultivations.

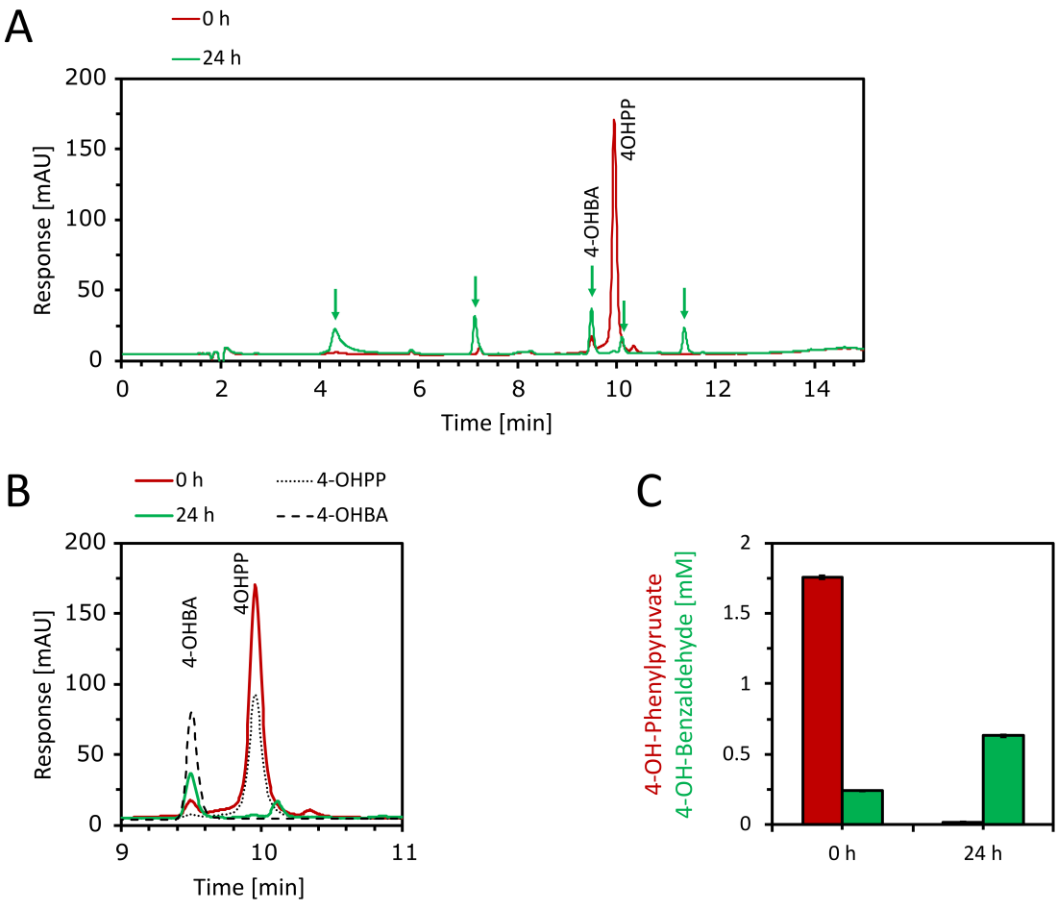

Figure S5: Degradation of 4-OH-phenylpyruvate in sterile CGXII minimal medium under regular cultivation conditions. (**A**) HPLC chromatogram overlays (detection with DAD at 304 nm) are shown for the samples after 0 h (red) and 24 h (green), as well as a zoom towards the retention time 9-11 mins (**B**), including the chromatograms of equimolar standards of 4-OH-benzaldehyde (4-OHBA; dashed line) and 4-OH-phenylpyruvate (4-OHPP; dotted line).

(**C**) Quantification of 4-OH-phenylpyruvate and 4-OH-benzaldehyde after 0 h and 24 h. Values and error bars represent means and standard deviations of triplicates.

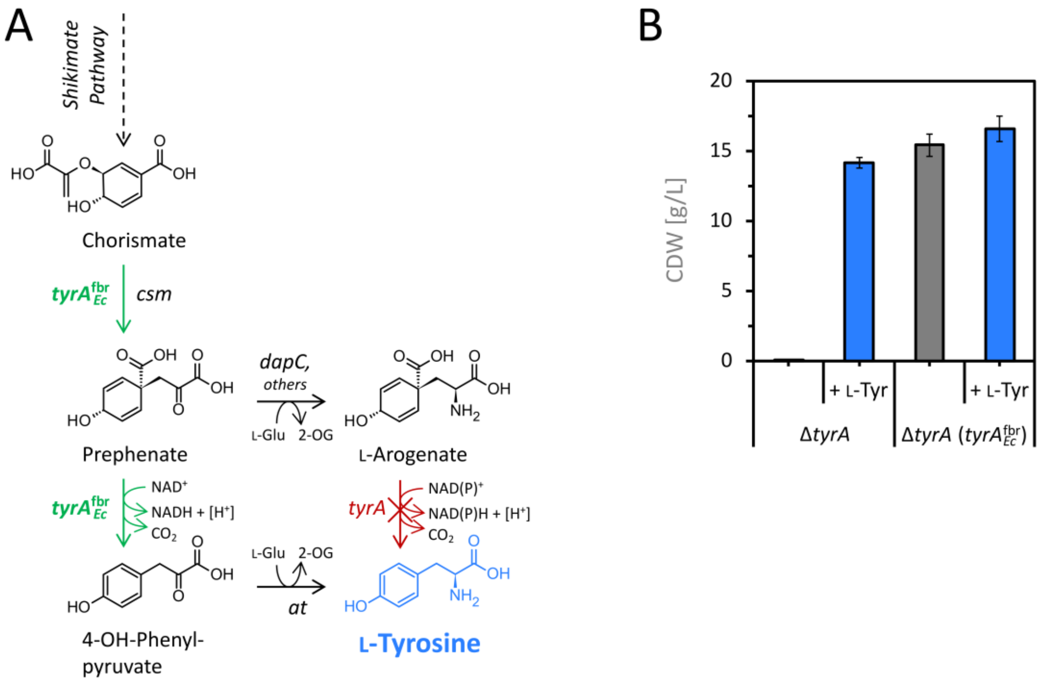

Figure S6: L-Tyrosine auxotrophy of the L-arogenate dehydrogenase deficient knockout strain AROM3 Δ*tyrA* was complemented by expression of the bifunctional chorismate mutase/prephenate dehydrogenase gene $tyrA_{Ec}^{fbr}$.

(**A**) L-Tyrosine biosynthesis pathway in *C. glutamicum* with deletion of *tyrA* (red) and overexpression of the heterologous gene $tyrA_{Ec}^{fbr}$ from *E. coli* (green).

(**B**) CDW after 48 h of cultivation of *C. glutamicum* Δ*tyrA* and Δ*tyrA* ($tyrA_{Ec}^{fbr}$) with or without supplementation of 1 mM L-tyrosine. Values and error bars represent means and standard deviations of triplicate cultivations.

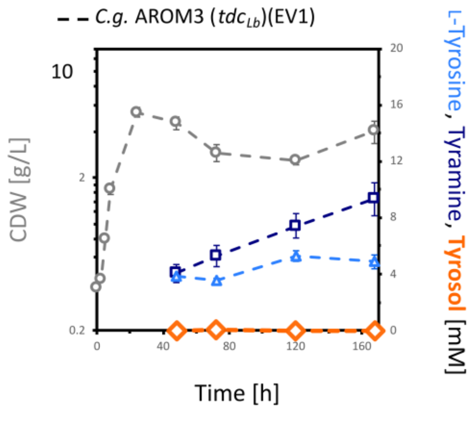

Figure S7: L-Tyrosine and tyramine production with plasmid-based expression of tdc_{Lb} in the empty vector carrying strain *C. glutamicum* AROM3. Values and error bars represent means and standard deviations of triplicate cultivations.

Table S1: Results of the protein BLAST search (1) of the 4-OH-phenylacetaldehyde-accepting alcohol dehydrogenases $Yahk_{Ec}$ (UniProt: P75691) and $ADH6_{Sc}$ (UniProt: Q04894) against the proteins encoded in the genome of *C. glutamicum*.

Accession	Per. Ident. to $YahK_{Ec}$	Per. Ident. to $ADH6_{Sc}$	Cg number	Description	Function	Ref-
**WP_011013571.1**	46.31	37.5	cg0400	alcohol dehydrogenase FudC	Reduction of furfural/ 4-formylphenol, protocatechuic aldehyde, and vanillin to the respective alcohol	(2)
**WP_011013485.1**	30.99	25.44	cg0273	Putative alcohol dehydrogenase	Unknown	(3)
**WP_011015397.1**	33.33	28.13	cg3107	Alcohol dehydrogenase AdhA	Oxidation of methanol to formaldehyde	(3)
**WP_003863269.1**	27.53	25.29	cg0387	NAD-linked mycothiol-dependent formaldehyde dehydrogenase	Oxidation of formaldehyde	(4)
**WP_011015614.1**	30.77	< 30	cg3405	NADPH:quinone reductase	Unknown	(5)
**WP_011265496.1**	< 30	31.15	cg0251	NADPH:quinone reductase/ related Zn-dependent oxidoreductase	Unknown	(6)

Table S2: Gene sequences used in this work.

Gene name	Sequence
**codon harmonized *ARO10*$_{Sc}$**	ATGGCACCAGTGACTATCGAAAAATTTGTTAACCAGGAAGAAAGGCATCTTGTGAGCAATAGGTCTGCAACTATCCCCTTCG<br>GCGAATATATTTTCAAGCGCCTGCTGAGCATTGATACGAAGTCTGTGTTTGGCGTGCCAGGCGACTTTAATCTGTCCCTTCTG<br>GAATACCTATACTCTCCAAGCGTGGAATCTGCAGGACTTCGCTGGGTTGGAACGTGCAACGAACTAAATGCGGCATACGCG<br>GCGGACGGATACAGCCGATATTCCAACAAAATCGGATGCCTGATTACTACGTACGGAGTGGGCGAACTGTCGGCGCTGAAT<br>GGCATTGCGGGCAGTTTTGCAGAAAACGTTAAGGTGCTGCATATCGTGGGCGTTGCGAAAAGCATTGATAGTCGATCTAGCA<br>ATTTCAGCGATAGAAATCTTCACCACCTGGTTCCACAACTTCACGATTCTAACTTCAAGGGGCCAAACCACAAGGTTTACCA<br>CGATATGGTTAAGGATCGCGTTGCATGTAGTGTTGCGTATCTGGAGGATATCGAAACCGCATGCGACCAGGTTGATAACGTG<br>ATTAGGGATATCTATAAATACTCCAAGCCAGGCTACATCTTCGTGCCAGCAGATTTCGCGGATATGTCCGTGACTTGCGATAA<br>CCTGGTGAACGTGCCACGAATTTCCCAGCAGGATTGCATTGTTTATCCATCCGAAAATCAGCTGTCCGACATTATTAATAAAA<br>TCACCAGCTGGATTTACAGCAGCAAGACTCCAGCGATTCTTGGAGACGTTCTAACCGATCGTTACGGCGTTAGCAATTTCCT<br>GAATAAACTTATTTGTAAGACCGGGATCTGGAACTTCAGCACCGTGATGGGAAAGTCCGTTATCGATGAGTCTAATCCAACC<br>TACATGGGCCAGTACAACGGCAAGGAAGGCCTGAAGCAGGTTTACGAACACTTCGAACTATGTGACCTGGTTCTGCACTTC<br>GGAGTTGACATTAACGAAATCAACAACGGGCACTACACCTTCACCTACAAGCCAAACGCAAAGATTATCCAGTTCCACCCC<br>AACTACATCAGGCTTGTTGACACCCGTCAAGGAAACGAGCAGATGTTTAAGGGAATTAACTTCGCGCCAATCCTGAAGGAA<br>CTTTATAAAAGGATCGACGTGTCCAAGCTTTCCCTGCAGTACGATTCTAACGTTACCCAGTACACGAATGAAACTATGAGAC<br>TGGAAGATCCAACTAACGGACAGTCTTCGATCATCACTCAGGTGCATCTGCAGAAAACGATGCCAAAGTTCCTGAATCCAG<br>GCGATGTGGTTGTGTGCGAAACTGGATCCTTCCAGTTTTCCGTGCGAGATTTTGCGTTCCCAAGTCAGCTGAAGTACATTAG<br>TCAGGGATTCTTTCTTAGCATCGGAATGGCGCTTCCAGCGGCGCTTGGCGTGGGAATCGCGATGCAGGACCATTCTAATGCA<br>CATATTAACGGCGGAAATGTTAAGGAGGACTACAAACCACGCCTGATCCTGTTCGAAGGCGACGGCGCAGCACAAATGACT<br>ATTCAGGAACTATCGACTATCCTAAAATGTAACATCCCACTTGAAGTGATTATCTGGAATAACAATGGATATACCATCGAACG<br>CGCGATTATGGGACCAACTCGTAGTTACAATGACGTGATGTCCTGGAAGTGGACTAAGCTTTTCGAAGCATTTGGAGACTTT<br>GACGGAAAATACACCAACTCGACCCTAATCCAGTGCCCCTCCAAGCTGGCACTAAAGCTGGAGGAGCTTAAAAACTCTAAT<br>AAGCGCTCGGGGATTGAACTTCTGGAAGTTAAGCTGGGAGAACTGGATTTTCCCGAACAACTTAAATGTATGGTGGAAGCA<br>GCGGCACTTAAGCGCAACAAGAAGTGA
***tyo*$_{Kr}$**	ATGAGCAACCCGCATGTCGTCGTGGTCGGAGCCGGATTCGCCGGGCTCGTGGCAGCCCGTGAGCTGCAGATGGCCGGCGT<br>GGACGTGGAGATCGTCGAGGCCCGGGACCGCGTGGGCGGCCGAGCATGGACCGAGGAGTGCATGGGACGCCCCCTCGAG<br>CTGGGGGCCACCTGGGTGCACTGGATGCAGCCGCACGTGTGGAGCGAGATCACCCGCTACGACCAGAGCATCTATCCCAGC<br>CCGTTCTGCGACGATGCCTACTGGATCACCGGCGACCGGGTGAAACACGGCACCGAGGCGGACCTCGACGCCGCACTCGC<br>CCGGCCCATGGCGAAGATCTTCGAGAACTCCCGCGAGTTCTTCCCCTATCCGTACGAACCCCTGCACGTCCTGGACGAGCG<br>CAGCGGCTCCTCTCCAGAGCTCCGGGAGAAGTTCCTGGCGGCGGACCGGGGCAGCGTCCTCGACTGCCTCCGGGGCGACG<br>AGTTCTCGCAGGAGGAACGGGACCTCGCCGACGCCTACTGGTCCGCGGCGTACATCGGGGACCCCCACAACGGCTCACCC<br>CTGATGGCCAAGCAGTGGGCGGCCCTCTCGGACCACCGGCTCTCCCTCGTGGACGAGCAGACGCTGCGGTTCAAGCTGAC<br>CCACGGCATGCGCGGGCTGTACGAGAACATCGCCGCAGACCTGCGTTGCCCCATTCGTCTGAACACGCCCGTGACCGCCAT<br>CGACCACCGTCATGACGGCGCGACCGTGACTCTCGGGACGGGGGAGAAGATCTCCTGCGACAGCGTGATCGTCACGGTTC<br>CGGTGGGAGCGCTGCCCAGCATCGACTTCACTCCCGCACTGCCCGCGGGGATGCGCAGCGTGGTCGACCAGAAGTGGAAC<br>TCCACCGGGTGCAAGATCTGGATCAAGGTCAAGGGCCACCACAGCGTCATCGGCTACGCCCCCACCCCGCACAAGGTGGC<br>CGTGTTCCGCAGCGAGTTCTTCATGGACGACGACACCACGATCTGCGTGGGCTTCGGCTCCCACCACGACGCAGTGGACCT<br>CACCGATCCTCGCGACGGCCAGGCCATCGTGGACCAGTGGCGCCCGGACCTGGAGGTGGTGGAGTGCACCGGGCACGACT<br>GGGTGGCGGACAAGTGGAGCGGCCAGGCATGGGCCACGTTGCGCTCCGGGCAGTTCACCAACGGGTGGCACCACTTCCGC<br>ACCACCGACTCCCGCCTGCGCTTCGCGGGCGCGGACTGGGCGCGCGGGTGGCGCGGCGTGGTGGTGGACGGGGCCATCGA<br>GACGGGTCTCTCCACTGCCCGGGAGGTCCTCAAGGACATCCGCGCCTGA

Table S3: Oligonucleotides used in this work.

Purpose	Primer name	Sequence (5' → 3')
Gibson assembly cloning of *tyrA*$_{Ec}$^{fbr} into pVWEx4	fw_tyrA_pVWEx4	GCCTGCAGGTCGACTCTAGAGCTTGACACAATTTGTATTGAAAGAAAAAGGAGGTTTTTTATGGTTGCTGAATTG
	rv_tyrA_M53I	GAGGCCAAAATAGATGCCTCGCGCTCCGGAACATAAATAGGCAGT
	fw_tyrA_M53I	GCGAGGCATCTATTTTGGCCTCGCGTCGTGCAGAGGCGGAAGC
	rv_tyrA_A354V	GAAAACGCTGGACGTAATCGCCGAACCAGTGCTCCACCTTGCGGA
	fw_tyrA_A354V	GGCGATTACGTCCAGCGTTTTCAGAGTGAAAGCCGCGTGTTATTGCG
	rv_tyrA_pVWEx4	GTGAATTCGAGCTCGGTACCCGGGGATCTTACTGGCGATTGTCATTCG
Sequencing of *tyrA*$_{Ec}$^{fbr}	fw_tyrA_Seq1	GGAATTGTGAGCGGATAACAATTTCACACAGG
	rv_tyrA_Seq2	GAATCCGCACCTGATAACCCGAGAGGG
	fw_tyrA_Seq3	GGGTTATCAGGTGCGGATTCTGGAGC
	fw_tyrA_Seq4	GCACGATCAGAATATGGCGTTTATTCAGGCACTGC
Sequencing of *ARO10*$_{Sc}$	fw_ARO10_Seq1	GCCTGATTACTACGTACGGAGTG
	fw_ARO10_Seq2	CCAGCGATTCTTGGAGAC
	rv_ARO10_Seq3	GTCATTTGTGCTGCGC
Gibson assembly cloning of *fudC* into pVWEx1	fw_0324_pVWEx1_1	CTAGAGGAGTATAATTCCTAAAAATAGAAGAAAAGGAGGTTAGTATGAGTATCTCAGTAAAAGCACTACAAAAGTCCGG
	fw_0324_pVWEx1_2	CGCCAAGCTTGCATGCCTGCAGGTCGACTCTAGAGGAGTATAATTCCTAAAAATAGAAGAAAAGGAGGTTAG
	rv_0324_pVWEx1	GTGAATTCGAGCTCGGTACCCGGGGATCCCTAAACCGCCTCAACCTCAGCAAACG
Sequencing of *fudC*	rv_0324_Seq1	CGCCTTCTTTAACGTTCC
	fw_0324_Seq2	CCATCACCCAAGGCGGC
	fw_0324_Seq3	CGCACGCTTGCGACTTC
Gibson assembly cloning of *tyo*$_{Kr}$ into pVWEx4	fw_tyo_pVWEx4	CTGCAGGTCGACTCTAGAGAGAGAACCAAGGTTATTAAAGGAGGTATTTTATGAGCAACCCGCATGTCG
	rv_tyo_pVWEx4	CTCGGTACCCGGGGATCTCAGGCGCGGATGTCC
Sequencing of *tyo*$_{Kr}$	tyo_seq1	GTGAGCGGATAACAATTTCACACAGG
	tyo_seq2	GGCCCTTGACCTTGATCCAG
	tyo_seq3	TGCCACCCGTTGGTGAACTG
	tyo_seq4	CGCTACTGCCGCCAGGCAAATTCTG
Gibson assembly	fw_ARO10Sc_pVWEx-tyoKr	CGCCAAGCTTGCATGCCTGCAGGTCGACTAGTAGTACGACTTGTAGGGTCGGG

cloning of *ARO10*$_{Sc}$ into pVWEx4-*tyo*$_{Kr}$	rv_ARO10Sc_pVWEx1-tyoKr	CCTTTAATAACCTTGGTTCTCTCTCTAGTCACTTCTTGTTGCGCTTAAGTGCCGC
Sequencing of chromosomal deletion of *csm*	fw_Delta-csm_Upstream	CAGGTCGACTCTAGAGGATCAACGTACGCGCCGAGAACTTC
	rv_Delta-csm_Downstream	GAGCTCGGTACCCGGGGATCCTGGTGGCGGTGAGGTATTC
Sequencing of chromosomal deletion of *dapC*	fw_Delta-dapC_Upstream	ACCCGGATCACTTTGCCTGG
	rv_Delta- dapC_Downstream	CCACGACGACCTTGGACTTC
Sequencing of chromosomal exchange of *qsuABCD*::P$_{tuf}$-*qsuC*	US-qsuA-rv	CCTGCAGGTCGACTCTAGAGGTTGGCAGCGCAACCAGTC
	qsu_deletion_fw	GTTCGTGGACAAGTGTGGTGG
	qsu_deletion_rv	CTACCGCGCGGATTAAACC
	DS-qsuD_fw	CTCAAAAAGTAGAAGAGTCAGTAAAACCTCGACGC
	Ptuf-qsuC_fw	CGATGAACAGGATCGACAGCGTTTTAGCGTGTCAGTAG
	qsuC_rv	GTCGAGGTTTTACTGACTCTTCTACTTTTTGAGATTTGCCAGG
Sequencing of chromosomal deletion of *fudC*	P-C23	CGACGGTGGCATTGTCTTG
	P-C24	TGTAAGGCTTTCCAAGCGGT
	fw_0324_Seq1	GCCAATGTTGGATCCGGTGAGGACTTTTC
	rv_0324_Seq2	GTCAACGAACGTTTCCTGTGCAG

References

1. Johnson M, Zaretskaya I, Raytselis Y, Merezhuk Y, McGinnis S, Madden TL. NCBI BLAST: a better web interface. Nucleic Acids Res 2008; 36(Web Server issue):W5-9.

2. Kim H-S, Choi J-A, Kim B-Y, Ferrer L, Choi J-M, Wendisch VF et al. Engineered *Corynebacterium glutamicum* as the Platform for the Production of Aromatic Aldehydes. Front Bioeng Biotechnol 2022; 10:880277.

3. Witthoff S, Mühlroth A, Marienhagen J, Bott M. C1 metabolism in *Corynebacterium glutamicum*: an endogenous pathway for oxidation of methanol to carbon dioxide. Appl Environ Microbiol 2013; 79(22):6974–83.

4. Lessmeier L, Hoefener M, Wendisch VF. Formaldehyde degradation in *Corynebacterium glutamicum* involves acetaldehyde dehydrogenase and mycothiol-dependent formaldehyde dehydrogenase. Microbiology (Reading) 2013; 159(Pt 12):2651–62.

5. Busche T, Silar R, Pičmanová M, Pátek M, Kalinowski J. Transcriptional regulation of the operon encoding stress-responsive ECF sigma factor SigH and its anti-sigma factor RshA, and control of its regulatory network in *Corynebacterium glutamicum*. BMC Genomics 2012; 13:445.

6. Li Z, Liu J-Z. Transcriptomic Changes in Response to Putrescine Production in Metabolically Engineered *Corynebacterium glutamicum*. Front Microbiol 2017; 8:1987.
